# Control of locomotor speed, arousal, and hippocampal theta rhythms by the nucleus incertus

**DOI:** 10.1038/s41467-019-14116-y

**Published:** 2020-01-14

**Authors:** Lihui Lu, Yuqi Ren, Tao Yu, Zhixiang Liu, Sice Wang, Lubin Tan, Jiawei Zeng, Qiru Feng, Rui Lin, Yang Liu, Qingchun Guo, Minmin Luo

**Affiliations:** 1 School of Life Sciences, Tsinghua University, Beijing, 100084 China; 2 Tsinghua-Peking Joint Center for Life Sciences, Tsinghua University, Beijing, 100084 China; 3 National Institute of Biological Sciences (NIBS), Beijing, 102206 China; 4 School of Life Sciences, Peking University, Beijing, 100871 China; 5 Peking University-Tsinghua University-NIBS Joint Graduate Program, Beijing, 102206 China; 6 Chinese Institute for Brain Research, Beijing, 102206 China

**Keywords:** Motor control, Neural circuits

## Abstract

Navigation requires not only the execution of locomotor programs but also high arousal and real-time retrieval of spatial memory that is often associated with hippocampal theta oscillations. However, the neural circuits for coordinately controlling these important processes remain to be fully dissected. Here we show that the activity of the neuromedin B (NMB) neurons in the nucleus incertus (NI) is tightly correlated with mouse locomotor speed, arousal level, and hippocampal theta power. These processes are reversibly suppressed by optogenetic inhibition and rapidly promoted by optogenetic stimulation of NI NMB neurons. These neurons form reciprocal connections with several subcortical areas associated with arousal, theta oscillation, and premotor processing. Their projections to multiple downstream stations regulate locomotion and hippocampal theta, with the projection to the medial septum being particularly important for promoting arousal. Therefore, NI NMB neurons functionally impact the neural circuit for navigation control according to particular brains states.

## Abstract (web summary)

In addition to activation of locomotor circuits, navigation also requires regulation of arousal and spatial memory processes. Here the authors identify neuromedin B neurons in the nucleus incertus and their subcortical projections in controlling these various processes during navigation.

## Introduction

The ability to move through an environment to find food, social partners, habitats, or to avoid danger is fundamental for an animal’s survival. When navigating at fast speed through a complex environment, an animal requires high arousal to sustain an attentive state and actively uses memory to update its awareness of environmental cues. Previous studies have provided many insights into the vertebrate neural circuits involved in locomotion, arousal, and memory. Locomotion requires rhythmic neuronal activity in the spinal cord, and is controlled by descending pathways consisting of a large network of interconnected brain areas, including the motor cortex, the basal ganglia, the hypothalamus, the mesencephalic locomotor region (MLR), and the cerebellum^1–4^. Regulation of arousal involves several hypothalamic nuclei, as well as the subcortical nuclei that send out ascending cholinergic and monoaminergic projections^5–8^. Regarding memory, it is well established that hippocampal theta rhythms, which are generated by brainstem–septohippocampal connections, become more pronounced during active memory integration^9–11^.

Locomotion, arousal, and theta rhythms are often linked, but it has been shown that they can be separately controlled^12–14^. It remains unknown how these behavioral and physiological processes are coordinately organized. Previous studies have hinted that the nucleus incertus (NI)—a brain region in the pontine brainstem—may be involved in some of these processes^15–18^, although the underlying cell-type-specific circuit mechanisms have not been elucidated. Located in the midline central gray of the dorsal pons, the NI contains intermingled GABAergic^19,20^ and glutamatergic^19,21^ neurons, and expresses several markers, including neuromedin B (NMB), relaxin-3 (Rln3), the type 1 corticotropin-releasing factor receptor (CRFR1), the D_2_-type dopamine receptor (D2R), and the orexin/hypocretin receptor^19,22–26^. A longstanding difficulty in studying the NI lies in experimentally separating the NI from adjacent regions like the lateral dorsal tegmental nucleus (LDT) and the dorsal raphe nucleus (DRN), which have also been implicated in arousal and/or locomotion^7,27^.

Here, we used a combination of mouse genetics, fiber photometry of Ca^2+^ signals, optogenetic activation and inhibition, transsynaptic circuit mapping, and electrophysiological recordings to shed light on the functional roles of NI neurons. We first generated the knockin mouse line *NMB-Cre* which allowed us to selectively record and manipulate the activity of NI neurons. We initially found that NI NMB neuronal activity is significantly correlated with locomotor speed, arousal levels, and hippocampal theta power. Moreover, these processes were suppressed by optogenetic inhibition and were facilitated by optogenetic stimulation of NI NMB neuron activity. Circuit mapping and electrophysiological recordings showed that NI NMB neurons integrate information associated with arousal and locomotor activity; moreover, they send out both GABAergic and glutamatergic projections to a number of brain areas associated with locomotion, arousal, and theta rhythms. Projection-specific optogenetic stimulation revealed multiple projections that are able to promote these behavioral processes. Projection-specific inhibition demonstrated that the control of arousal by NI NMB neurons requires predominantly the projection to the medial septum and that the control of locomotion and hippocampal theta likely requires the coordinated activity of multiple downstream projections.

## Results

### NI NMB neurons encode locomotion, arousal, and theta rhythms

**Fig. 1:**
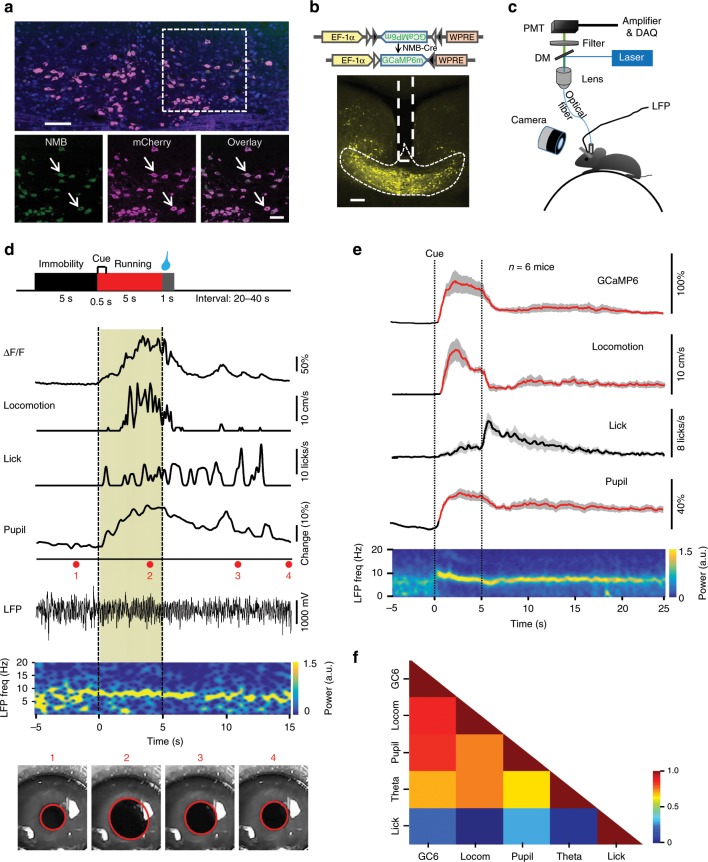
The activity of NI NMB neurons is correlated with locomotion, arousal, and theta power. **a** Up, images showing the colocalization of *NMB* mRNA (green), mCherry (red), and NeuN (blue) in the NI of an *NMB-Cre* mouse (98.4% NMB^+^ neurons expressed mCherry, *n* = 2284/2322 neurons; 89.0% mCherry^+^ neurons expressed NMB, *n* = 2284/2565 neurons, 24 sections from 4 mice), scale bars = 100 µm. NI NMB neurons were labeled with mCherry by injecting *AAV-DIO-mCherry* vectors into the NI of an NMB-Cre mouse. Bottom, the zoom-in view of the dashed rectangular area, scale bars = 50 µm. **b** Expressing GCaMP6m in NI NMB neurons. Scale bars = 200 µm. **c** The method of simultaneously monitoring GCaMP signals, animal locomotion, arousal, and hippocampal local field potentials (LFP) from a head-fixed mouse running on a wheel treadmill. DM, dichroic mirror; PMT, photomultiplier tube. An infrared camera was used to measure pupil diameter as a proxy of arousal. **d** Behavior paradigm and example data from one experimental trial. LFP signals are shown as a raw bandpassed signals (0.1–200 Hz), together with the 1–20 Hz spectrogram. Video frame images of the mouse’s eye (1–4) are shown where acquired at the times indicated in the pupil recording trace. Pupil diameter was extracted posthoc via a fitted ellipse (red). The left dashed line indicates the onset of cue and the right dashed line indicates the onset of liquid reward. **e** The group data of GCaMP signals, the locomotor speed, the normalized pupil diameter, lick signal, and theta rhythm spectrogram (*n* = 6 mice). Red segments indicate statistically significant increase from the baseline (*P* < 0.01; multivariate permutation test). Shaded areas indicate SEM. **f** Cross-correlation analysis of GCaMP signal, locomotor speed (locom), normalized pupil diameter, theta power change, and lick rates. Color scale to the right indicates correlation values.

Taking advantage of the previous finding that within the brainstem the neuromedin *B* (*NMB*) gene is preferentially expressed in the NI^19^, we used the CRISPR-Cas9 method to generate a knockin mouse line (*NMB-Cre*), in which the Cre recombinase is expressed under the control of the *NMB* promoter (Supplementary Fig. 1a). By infusing adeno-associated virus (AAV) vectors for Cre-dependent expression of red fluorescent protein [double-floxed inverted open reading frame (DIO)-mCherry], we were able to label neurons within the NI, without labeling surrounding brainstem areas (Fig. 1a). We quantified the specificity of *Cre* expression in NI neurons of *NMB*-Cre mice: in situ hybridization combined with virus expression showed that ~90% of mCherry-expressing cells in the NI were positive for *NMB* mRNA expression (Fig. 1a). It is possible that this number represents an underestimation, since some mCherry-expressing neurons may express *NMB* at a level below the detection limit of our in situ hybridization analysis yet at a level sufficient to drive Cre-dependent mCherry expression. Among mCherry-expressing NMB neurons, ~50% expressed Rln3 and ~37% expressed CRFR1 (Supplementary Fig. 1b, c). In an open field test, both heterozygous and homozygous mice exhibited grossly normal locomotor activity (Supplementary Fig. 1d–f). Thus, the *NMB*-Cre mouse line enables us to specifically express genetic probes in NI *NMB* neurons to dissect their functional roles.

Using changes in intracellular free Ca^2+^ levels as the indicator of neuronal activity, we examined the activity of NI NMB neurons in freely behaving mice. We expressed the Ca^2+^ indicator GCaMP6m and then applied fiber photometry to measure GCaMP fluorescence changes in NI NMB neurons (Fig. 1b). Given that NI neurons might be involved in stress response^24,28^, we first tested GCaMP6m-expressing mice in a Pavlovian conditioning paradigm, which featured delivery of an auditory cue followed by footshocks, quinine, or sucrose (see Methods). NI NMB neurons showed robust GCaMP fluorescence increase upon delivery of electric footshocks when such shocks caused immediate locomotion (Supplementary Fig. 2a). However, in trials that delivered quinine or sucrose but did not elicit locomotion, we did not observe significant GCaMP signals (Supplementary Fig. 2b, c). When mice moved freely in an open field, the mean GCaMP signals of NI NMB neurons were significantly higher during locomotion than during resting phases (Supplementary Fig. 2d, e), and the slope of the rising phase of the GCaMP signals was positively correlated with locomotor speed (Supplementary Fig. 2f). These results suggest that the activity of NI NMB neurons corresponds to locomotion, rather than stress or reward per se.

We next designed a head-fixed locomotion task to carefully test how the activity of NI NMB neurons is associated with locomotor speed as well as arousal and hippocampal theta rhythms. We trained water-restricted mice to run in response to an odor cue: a drop of sucrose solution was given if mice ran for more than 3 s within the 5 s time window following the cue onset (Fig. 1c, d). In recording GCaMP signals using fiber photometry, we simultaneously used an infrared camera to measure pupil size to monitor arousal^8,14,29,30^, and used a tungsten microelectrode to record local field potentials (LFP) in the hippocampal CA1 (Fig. 1c, d and Supplementary Fig. 2g, h). Aligning the GCaMP signals with cue onset revealed an increase in the activity of NI NMB neurons starting at the onset of locomotor acceleration (Fig. 1d, e; Supplementary Fig. 2i, j; Supplementary Movie 1). The GCaMP signal data from a time course experiment were strongly correlated (Pearson’s *r*) with the data for locomotor speed, pupil dynamics, and theta power (Fig. 1f and Supplementary Fig. 2k).

Given that locomotion is often closely associated with arousal and hippocampal theta^12–14^, we asked whether NI NMB neurons could be activated following heightened arousal but in the absence of locomotion. To dissociate arousal from locomotion, we induced a state of enhanced arousal by puffing air on the back of head-fixed mice^14^ (see Methods). In a subset of trials, delivery of airpuff increased arousal levels (15% increase in pupil diameter) and decreased theta power (23%), but did not induce locomotive activity. Across these trials, airpuff resulted in a mild but significant increase (~7.1%) in GCaMP signals that was positively correlated with arousal levels (*r* = 0.66; Supplementary Fig. 3a, b). We did not observe any clear changes in fluorescence when airpuff were applied to control mice that expressed GFP in the NI neurons (Supplementary Fig. 3c) or when the GFP-expressing mice moved during the reward-retrieval task (Supplementary Fig. 3d, e), confirming that the GCaMP6 fluorescence changes represented Ca^2+^ signals rather than artefacts of animal movement. Together, these results indicate that the activity of NI NMB neurons is strongly associated with locomotion, arousal, and hippocampal theta oscillation. Moreover, the activity of these neurons is also positively correlated with arousal levels, even in the absence of locomotion.

### Effects of optogenetical inhibition of NI NMB neurons

**Fig. 2:**
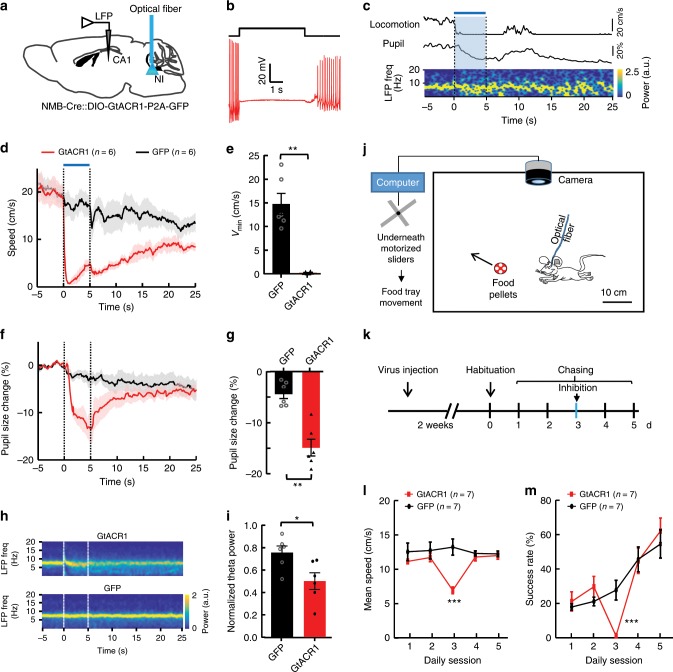
Inhibiting NI NMB neurons suppresses locomotion, arousal, and theta power. **a** Experimental schematic for inhibiting NI NMB neurons and recording hippocampal LFP in head-fixed mice. **b** A blue laser pulse (5 s) abolished action potential firing elicited by current injection into a GtACR1-expressing neuron in the NI (*n* = 7 cells tested). **c** Raw traces showing the effect of optogenetic inhibition (blue bar) of NI NMB neurons on locomotion, arousal, and theta rhythms. **d** Locomotor speed aligned to laser onset. **e** Bar plot showing the inhibition effects on locomotor speed (*n* = 6 mice for all groups). **f**, **g** The effect of optogenetic inhibition on the pupil diameter across time (**f**) and summary data (**g**). **h**, **i** Grand mean of hippocampal LFP spectrograms aligned to laser onset (**h**) and summary data of optogenetic inhibition on theta power normalized to the sum of 0.1–12 Hz power (**i**). **j**, **k** Schematic (**j**) and time line (**k**) illustrating the food-chasing task, in which a mouse chased after a moving food tray to retrieve food pellets. In the third behavioral session we optogenetically inhibited NI neurons (**k**). **l**, **m** Optogenetic inhibition of NI neurons suppressed the chasing speed (**l**) and aborted the success rate of reaching the moving food tray (**m**). Shaded areas (**d**, **f**) and error bars (**e**, **g**, **i**, **l**, **m**) indicate SEM. *P < 0.05, **P < 0.01, ***P < 0.001; unpaired *t* test; see Supplementary Table 1 for detailed statistical analysis. Source data are provided as a Source Data file.

Next, we examined how inhibiting the activity of NI NMB neurons acutely affects mouse behavior. Injecting *AAV-DIO-GtACR1-P2A-GFP* vectors into the NI of *NMB-Cre* mice led to the expression of the light-sensitive chloride channel GtACR1, which, upon light illumination, immediately suppressed the firing of action potentials in NI NMB neurons (Fig. 2a, b; Supplementary Fig. 4a, b). We then implanted an optical fiber to examine the effects of optogenetic inhibition of NI neurons in head-fixed animals (Fig. 2a; Supplementary Fig. 4c). Interestingly, optical inhibition caused running animals to decelerate rapidly, often to a full stop, whereas control GFP-expressing mice with light delivery showed only a gradual decrease in mean speed over time (Fig. 2c–e; Supplementary Fig. 4d–g; Supplementary Movie 2). Moreover, compared to baseline, optogenetic inhibition of NI NMB neurons caused a significant decrease in normalized pupil diameter (inhibition, ~15%; control, ~4%; Fig. 2f, g). During light delivery the GtACR1-expressing mice exhibited desynchronized LFP in the hippocampus and thus significantly weaker theta power than control mice (Fig. 2h, i). In immobile mice that received airpuff at the back but did not exhibit an increase in locomotion, optogenetic inhibition of NI NMB neurons reduced pupil diameter but did not affect hippocampal theta power (Supplementary Fig. 4h–l). Collectively, our findings establish that the activity of NI NMB neurons is required for maintaining locomotion, arousal, and high theta power.

Next we tested whether or not NI NMB neurons play a role in appetitive locomotion in freely moving animals. We trained GCaMP6m-expressing mice in an open field to obtain food pellets by chasing after a moving food tray (Fig. 2j). Fiber photometry revealed significant correlations between Ca^2+^ signals and the locomotor speed data from the food-chasing task (Supplementary Fig. 5a–d). We then delivered light into the NI of GtACR1-expressing mice or GFP-expressing control mice when a mouse initiated its chasing of the food tray (Fig. 2j, k). Optogenetic inhibition slowed down locomotion speed by 50%, and nearly abolished an animal’s ability to successfully collect food from the moving food tray (Fig. 2l, m; Supplementary Movie 3). Despite the optical inhibition-induced failures, mice continued to make efforts to initiate chasing (failure/total trials in the control group: 47/61; in the experiment group: 99/101; Supplementary Fig. 5e–h). These results thus show that the activity of NI NMB neurons is required for appetitive locomotion. Moreover, inactivating NI neurons disrupts locomotor speed control but does not reduce the motivation for reward seeking.

### Effects of optogenetic activation of NI NMB neurons

**Fig. 3:**
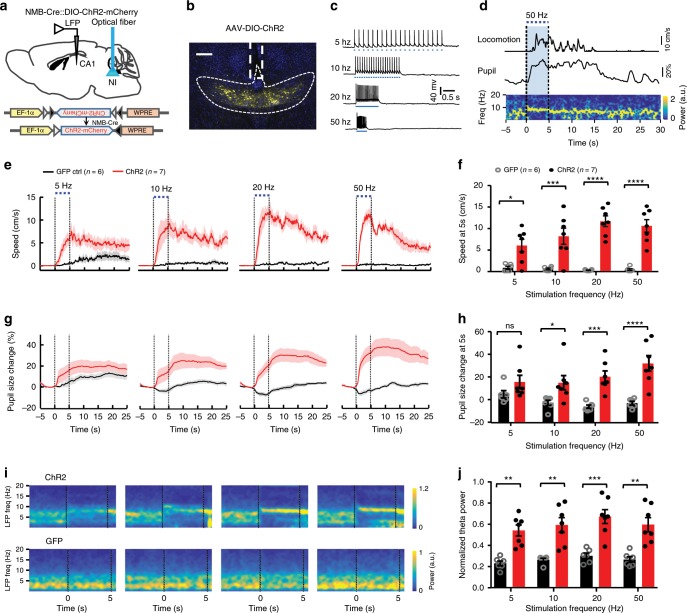
Activating NI NMB neurons promotes locomotion, arousal, and theta power. **a** Experimental schematic for viral expression of ChR2, stimulating NI NMB neurons, and recording hippocampal LFP. **b** A coronal section shows ChR2-mCherry expression in NI NMB neurons. Scale bar = 200 µm. **c** Recording from brain slices confirmed that brief blue laser pulses at 5, 10, 20 and 50 Hz (5 ms width, 5 mW) reliably elicited the firing of action potentials in a ChR2-expressing NI neuron. **d** A representative example showing the effect of optogenetic activation of NI NMB neurons (blue bar) on inducing locomotion, arousal, and theta oscillations. **e** Locomotor speed aligned to different frequency laser onset. **f** Summary of the stimulation effects on locomotor speed. **g**, **h** The effect of optogenetic activation of NI NMB neurons on the pupil diameter across time (**g**) and summary data (**h**). **i**, **j** Grand average of LFP spectrograms for the entire test group (**i**) and summary data on the theta power (**j**). Shaded areas (**e**, **g**) and error bars (**f**, **h**, **j**) indicate SEM. *P *<* 0.05, **P *<* 0.01, ***P *<* 0.001, ****P *<* 0.0001; ns, not significant; Tukey’s multiple comparisons test; see Supplementary Table 1 for detailed statistical analysis. Source data are provided as a Source Data file.

We then investigated the functional effects of activating NI NMB neurons. We expressed the light-sensitive cation channel ChannelRhodopsin-2 (ChR2) in NI NMB neurons following the infusion of *AAV-DIO-ChR2-mCherry* vectors into the NI of *NMB-Cre* mice (Fig. 3a, b; Supplementary Fig. 6a). Whole-cell recordings from brain slices confirmed that shining tonic pulse trains (20 pulses, 5 ms width at 5, 10, 20, or 50 Hz) reliably activated ChR2-expressing NI neurons (Fig. 3c; Supplementary Fig. 6b). We then implanted an optical fiber to deliver trains of light pulses of various frequencies into the NI of head-fixed mice. Optical activation of NI NMB neurons reliably produced a time-locked increase in the locomotor speed, the pupil diameter, and the theta rhythm power of these mice (Fig. 3d and Supplementary Movie 4). Stimulation at 5 Hz produced small but statistically significant increases, and those at higher frequencies (10, 20, 50 Hz) led to greater increases in locomotor speed and pupil diameter, with the effects reaching saturation at 20 Hz (Fig. 3e–h). The locomotor speed peaked at the end of stimulation and then gradually decreased over time (Fig. 3e, f). Optogenetic stimulation of the NI NMB neurons also increased hippocampal theta power (Fig. 3i, j; Supplementary Fig. 6c). Such stimulation promoted locomotor activity in freely behaving mice in an open field (Supplementary Fig. 6d–h), which indicated a stimulatory effect in different behavioral states. As a control, no significant changes in locomotor speed, pupil size, or theta power were observed in mCherry-expressing mice (Fig. 3e–j). In sum, tonic stimulation of NI NMB neurons accelerates locomotion, increases arousal, and strengthens hippocampal theta rhythms.

**Fig. 4:**
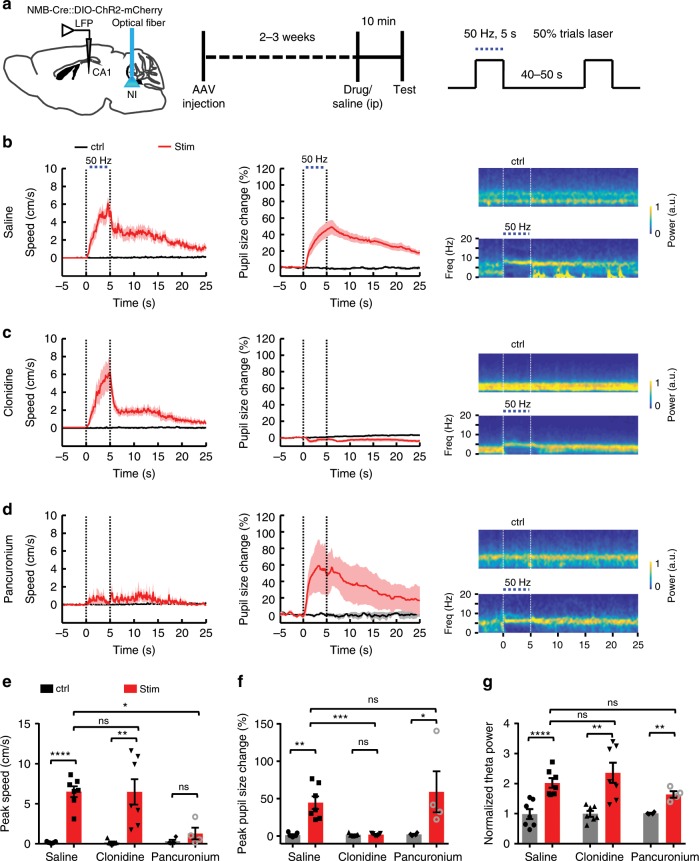
The effect of NI NMB neurons on locomotion, arousal, and theta power are decomposable. **a** Schematics show the method of optogenetic stimulation of NI NMB neurons (left), time line of behavior test (middle), and stimulation protocol (right). **b** The effect of optogenetic stimulation on average locomotor speed (left), pupil diameter change (middle), and grand average of LFP spectrograms (right) for NMB-NI^ChR2^ mice with saline control injection (ip; *n* = 7 mice). **c** The effect of applying the adrenergic alpha-2 receptor agonist clonidine (0.1 mg kg^−1^, ip) of NI NMB neurons (*n* = 7 mice). **d** The effect of applying the muscle relaxant pancuronium (0.18 mg kg^−1^, ip; *n* = 4 mice). **e**–**g** Summary data of showing the effect of clonidine and pancuronium on locomotor speed change, pupil diameter change, and theta power change in response to optogenetic stimulation of NI NMB neurons. Shaded areas (**b**–**d**) and error bars (**e**–**g**) indicate SEM. *P *<* 0.05, **P *<* 0.01, ***P *<* 0.001, ****P *<* 0.0001; ns, not significant; Paired *t* test and Mann–Whitney test; see Supplement Table 1 for detailed statistical analysis. Source data are provided as a Source Data file.

Given that locomotion, arousal, and theta rhythms are often linked, we examined whether the stimulatory effects are dissociable. We first blocked arousal by applying clonidine (0.1 mg kg^−1^, ip), which reduces central norepinephrine levels and induces miosis^31–33^. In the presence of clonidine, stimulating NI NMB neurons failed to trigger pupil dilation but remained effective to promote locomotion and theta power (Fig. 4a–c, e–g), which suggested that the effect of NI activation on locomotion and theta oscillation is not secondary to heightened arousal. We then injected pancuronium (0.18 mg kg^−1^, ip), which blocks neuromuscular junction and thus prevented locomotion^34^. In the presence of pancuronium, stimulating NI NMB neurons failed to trigger locomotion acceleration but remained effective to enhance arousal and hippocampal theta power (Fig. 4d–g), which suggested that the stimulatory effect of NI NMB neurons on arousal and theta oscillation is not secondary to animal locomotion.

We also observed that optogenetic stimulation of NI NMB neurons increased the plasma levels of epinephrine—the hormone associated with arousal (Supplementary Fig. 7a). To test whether changing the activity of NI NMB neurons affected an animal’s emotional valence, we performed both conditioned place preference test and real-time place preference test. Neither stimulating nor inhibiting NI NMB neurons affected place preference or avoidance (Supplementary Fig. 7b, c), suggesting that the activity of these neurons does not signal direct rewarding or aversive quality.

### Presynaptic partners of NI NMB neurons

**Fig. 5:**
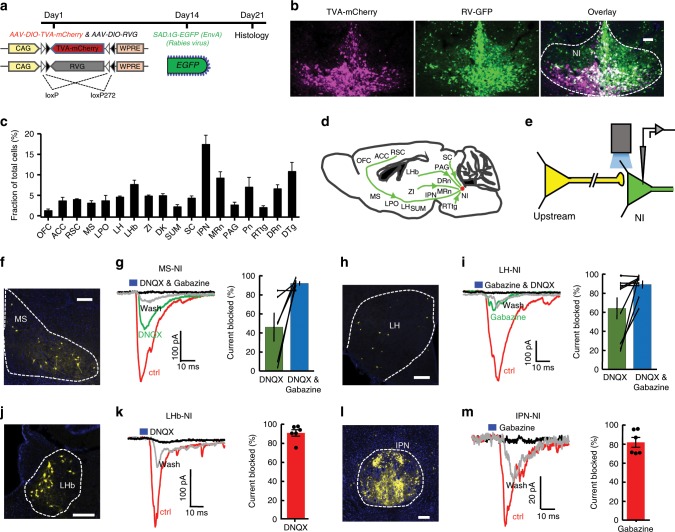
NI NMB neurons receive inputs from brain areas associated with arousal and locomotion. **a** The strategy for monosynaptic retrograde tracing of NI NMB neurons. **b** The expression pattern of TVA-mCherry (red) and RV-GFP (green) at the injection site within the NI. Dual labeled cells indicate starter cells competent for retrograde transsynaptic traversal. **c** Ratio of total retrogradely-labeled neurons in various upstream stations of NI NMB neurons (*n* = 4 mice). **d** Schematic showing the inputs of NI NMB neurons. **e** Schematic diagram showing terminal photostimulation of the upstream neurons and whole-cell patch recording of the NI NMB^+^ cells. **f** Presynaptic RV^+^ cells in the MS. **g** Postsynaptic responses from a NMB^+^ neuron in the NI following photostimulation of ChR2^+^ MS neuron axonal terminals under the conditions of control (latency, 8.1 ± 0.8 ms), drug applications, and wash. Postsynaptic current was measured holding at −65 mV. The right panel shows the summary effect of DNQX and the addition of Gabazine (*n* = 6 NMB^+^ cells from 2 mice). **h**, **i** Presynaptic RV^+^ cells in the LH (**h**) and the physiological effect of photostimulating ChR2-expressing LH axonal terminals on NI NMB neurons (**i**; latency, 6.6 ± 0.6 ms). Right panel in (**i**) shows the drug effects (*n* = 9 NMB^+^ cells from 2 mice). **j**, **k** Presynaptic RV^+^ cells in the LHb (**j**) and the physiological effect of activating LHb axonal terminals on NI NMB neurons (**k**; latency, 8.5 ± 1.0 ms; *n* = 6 NMB^+^ cells from 2 mice). **l**, **m** Presynaptic RV^+^ cells in the IPN (**l**) and the physiological effect of activating IPN axonal terminals on NI NMB neurons (**m**; latency, 6.2 ± 1.2 ms; *n* = 6 NMB^+^ cells from 2 mice). Error bars (**c**, **g**, **i**, **k**, **m**) indicate SEM. Scale bars = 200 µm (**b**, **f**, **h**, **j**, and **l**). Source data are provided as a Source Data file.

Next we asked how NI NMB neurons connect with brain regions associated with locomotion, arousal, and theta rhythms. To map presynaptic partners of NI NMB neurons, we conducted mono-transsynaptic retrograde tracing using recombinant rabies virus (RV) (Fig. 5a, b). We observed inputs mainly from subcortical areas associated with locomotor activity and motor control (e.g., the medial septal nucleus (MS), the zona incerta (ZI), the interpeduncular nucleus (IPN), the periaqueductal gray (PAG), the dorsal tegmental nucleus (DTg))^12,27,35–37^; arousal and brain states (e.g., the MS, the lateral hypothalamus (LH))^6,38^; and theta rhythms generation (e.g., the MS, the supramammillary nucleus (SUM), the median raphe nucleus (MRN))^12,39–42^. We also observed less dense inputs from other regions, including the lateral habenula (LHb), the DRN, the pontine reticular nucleus (Pn), the orbital cortex (OFC), the anterior cingulate cortex (ACC), and the retrosplenial cortex (RSC), which are consistent with previous literatures^18,43^ (Fig. 5c, d, f, h, j, l, Supplementary Fig. 8a). Finally, we observed inputs from the nucleus of Darkschewitsch (DK), the superior colliculus (SC), and reticular tegmental nucleus of the pons (RtTg) that have not previously been observed^43^ (Fig. 5c, Supplementary Fig. 8a). We did not observe labeling in the extra-NI areas of control mice that lacked components essential for RV infection or cross-synaptic jumping (Supplementary Fig. 8b–d), demonstrating the validity of using recombinant RV for retrograde transsynaptic labeling.

We next sought a proof-of-concept validation that our anatomical mapping corresponds to functional interactions. We optogenetically activated four brain regions known to send strong projections to the NI (the MS, the LH, the LHb, and the IPN) and determined which (if any) neurotransmitters their respective projections release into the NI. *AAV-EF1a-DIO-mGFP* was injected into the NI of *NMB-Cre* mice and a mixture of *AAV-hSyn-Cre* and *AAV-EF1a-DIO-ChR2-mCherry* (equal volumes) was injected to each of the upstream regions (separate mice for each of the four upstream regions) (Supplementary Fig. 8e). We prepared brain slices containing the NI and recorded from GFP-expressing neurons within the NI (Fig. 5e; Supplementary Fig. 8f–i). Optogenetic stimulation of MS ChR2-expresing terminals elicited postsynaptic responses in NI NMB neurons. The ionotropic glutamate receptor antagonist DNQX blocked 46.04% of responses to light stimulation, and the combined presence of DNQX and the GABA_A_ receptor antagonist Gabazine blocked 92.0% of these responses (Fig. 5g), thus suggesting that both glutamatergic and GABAergic neurons of the MS target NMB neurons in the NI. We also observed both glutamatergic and GABAergic components within the projection from the LH to the NI (Fig. 5i). Stimulation of LHb ChR2-expresing terminals elicited EPSCs in six NI NMB neurons (Fig. 5k). Stimulation of IPN ChR2-expresing terminals elicited pure IPSCs in 6/8 NI NMB neurons (Fig. 5m) and a mixture of EPSC and IPSC in 2/8 NI NMB neurons (Supplementary Fig. 8j). These responses were recorded in the presence of 4-AP and tetrodotoxin^44^, indicating that the neurons from the MS, the LH, the LHb, and the IPN each form functional monosynaptic connections to NI NMB neurons. Our results from these anatomical and electrophysiological experiments thus show that NI NMB neurons received both GABAergic and glutamatergic projections from the MS, the LH, and the IPN, and exclusively glutamatergic projections from the LHb.

### Projection targets of NI NMB neurons

**Fig. 6:**
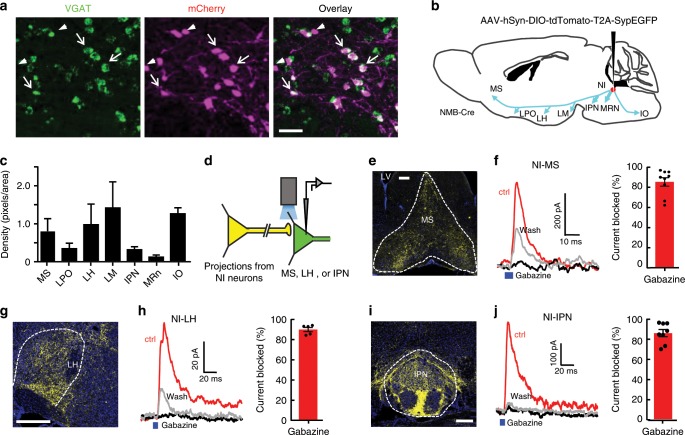
NI NMB neurons project to multiple brain areas. **a** The presence of *VGAT* (*slc32a1*) mRNA (green) in mCherry-expressing NI NMB neurons (*n* = 937 VAGT^+^/1234 mCherry^+^ neurons; 937 mCherry^+^ /2089 VGAT^+^ neurons; 20 sections from 4 mice). Arrows, dual labeled cells; Arrowheads, mCherry + cells that clearly lacked *VGAT* mRNA expression. **b** Schematic shows that expressing synaptophysin-EGFP in NI NMB neurons labels terminals in several subcortical structures. **c** The normalized density of innervations (total EGFP^+^ pixels divided by the area of each nucleus; *n* = 4 mice). **d** Schematic diagram showing the method of optogenetic stimulation and recordings from the MS, the LH or the IPN in brain slices. **e, f** Terminal expression of synaptophysin-EGFP in the MS (**e**) and the effect of activating ChR2-expressing terminals on evoking IPSCs from MS neurons (**f**). The left panel in (**f**) shows representative traces of light-evoked IPSCs from an MS neuron before (red; latency, 4.8 ± 1.1 ms), during (black), and after (gray) Gabazine application. The right panel shows the group data on the effect of Gabazine on blocking the light-evoked IPSCs (*n* = 9 cells from 4 mice). Postsynaptic current was measured holding at −10 mV. **g**, **h** Terminal projection pattern of NI NMB neurons in the LH (**g**) and the effects of activating NI NMB neurons on evoking IPSCs from LH neurons (**h**; latency, 3.9 ± 1.8 ms; *n* = 5 cells in 5 mice). **i**, **j** Distribution of NI NMB axonal terminals in the IPN (**i**) and the effects of activating the terminals on the Gabazine-sensitive IPSCs of IPN neurons (**j**; latency, 6.1 ± 0.5 ms; *n* = 8 cells from 5 mice). Error bars (**c**, **f**, **h**, **j**) indicate SEM. Scale bars = 50 µm (**a**), 200 µm (**e**, **g**, **i**). Source data are provided as a Source Data file.

We next explored how NI NMB neurons functionally regulate the activity of downstream neurons. Labeling with mCherry and in situ hybridization mapping of mRNA transcripts for the vesicular GABA transporter (*Vgat*) revealed that ~76% of NMB neurons are apparently GABAergic (Fig. 6a), suggesting that NI NMB neurons provide mainly GABAergic outputs. To visualize the terminal projection pattern, we performed anterograde tracing by targeting a Cre-dependent AAV expressing synaptophysin-EGFP to NI NMB neurons (Fig. 6b). This revealed dense projections from the NI to a number of brain regions known to be involved in controlling locomotor activity (e.g., the inferior olive nucleus (IO), the lateral mammillary nucleus (LM), the IPN, and the MRN)^36,40,45,46^; arousal (e.g., the LH and the lateral preoptic nucleus (LPO))^6,47^; and theta rhythms (e.g., the MS)^12,41,42^ (Fig. 6c, e, g, i; Supplementary Fig. 9a–d). No obvious axons were observed in the spinal cord.

To validate that our anatomical mapping corresponds to functional interactions, we chose three representative targets (the MS, the LH, and the IPN) to characterize neurotransmitters released by NI NMB neurons. We prepared brain slices containing these target regions from *NMB-Cre* mice expressing ChR2 in the NI, and performed patch-clamp recordings from neurons located at the terminals of NI projections in each target region (Fig. 6d; Supplementary Fig. 9e–g). Optical stimulation of ChR2-expresing projection terminals elicited fast GABA_A_-mediated inhibitory postsynaptic currents (IPSCs) in each of the target regions (MS, *n* = 9/9 cells with latency *=* 4.8 ± 1.1 ms; LH, *n* = 5/15 cells with latency *=* 3.9 ± 1.8 ms; IPN, *n* = 8/8 cells with latency *=* 6.1 ± 0.5 ms) (Fig. 6f, h, j). In 10/15 LH neurons, we also detected fast AMPA-mediated excitatory postsynaptic current (EPSC) responses with a short latency (3.8 ± 0.5 ms; Supplementary Fig. 9h). The synaptic responses were recorded in the presence of the sodium channel blocker tetrodotoxin and the potassium channel blocker 4-AP^44^, which again indicated that NI NMB neurons form monosynaptic connections to the MS, the LH, and the IPN. These results suggested that most of the NI NMB neurons are GABAergic but that some are glutamatergic; this is consistent with our in situ hybridization results (Fig. 6a). Our results from these anatomical and electrophysiological experiments thus show that NI NMB neurons provide both GABAergic and glutamatergic projections to target brain regions that are associated with locomotion, arousal, and theta rhythm generation. We compared the effects of stimulating NMB^+^ cells, GABA neurons, and glutamate neurons in the NI using *NMB-Cre* mice, *Vgat-Cre* mice, and *Vglut2-Cre* mice. We observed rather complex cell type-specific effects on different processes: for locomotion, NMB > glutamate > GABA = 0; for arousal, glutamate > NMB > GABA > 0; and finally for theta, NMB ≥ glutamate > GABA > 0 (Supplementary Fig. 10).

### Projection-specific roles in locomotion, arousal, and theta

**Fig. 7:**
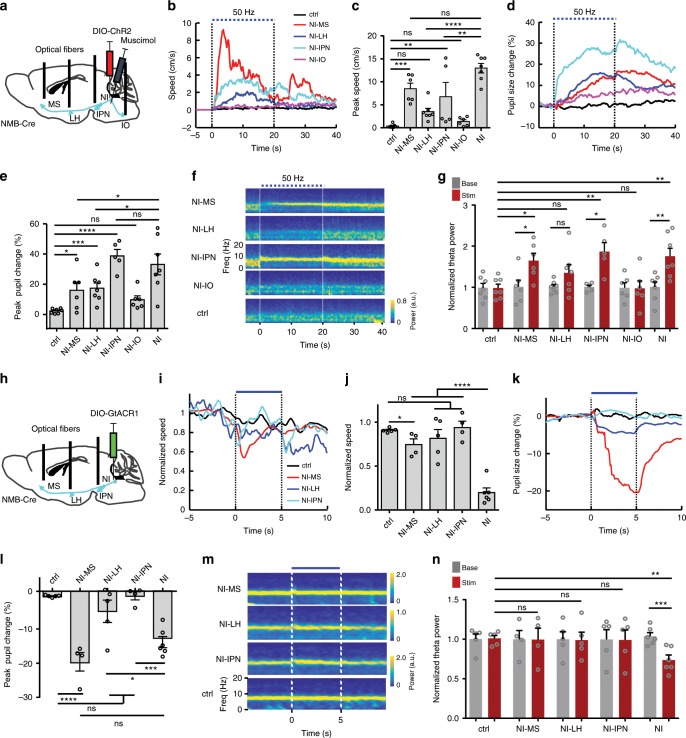
NI-MS projections differentially modulates locomotion, arousal, and theta power. **a** Experimental schematic for viral expression of ChR2 and stimulation of NI terminals at different target regions. **b, c** Average traces (**b**) and summary data (**c**) showing the locomotor speed change evoked by terminal stimulation in the MS (red; *n* = 5 mice), the LH (blue; *n* = 7 mice), the IPN (cyan; *n* = 5 mice), and the IO (magenta; *n* = 6 mice) as well as stimulation of soma area in the NI (*n* = 7 mice). Dashed blue line marks the duration of laser stimulation. **d, e** Average traces (**d**) and summary data (**e**) showing the change in normalized pupil diameter elicited by stimulating different projections and NI somata. **f, g** Grand average of LFP spectrograms (**f**) and summary data of theta power change (**g**) elicited by terminal stimulations in different target regions and the NI soma area (theta power is normalized to its base in each group). **h** Experimental schematic for viral expression of GtACR1 and optogenetic inhibition of NI terminals in various target regions. **i, j** Average normalized locomotor speed traces (**i**) and summary data showing the effect of optogenetically inhibiting axonal terminals in the MS (red; *n* = 4 mice), LH (blue; *n* = 5 mice), and IPN (cyan; *n* = 4 mice). Blue horizontal line and dashed vertical lines marks the duration of light inhibition. **k, l** Average traces (**k**) and summary data showing the effect of terminal inhibition and soma inhibition on pupil diameter. **m, n** Grand average of LFP spectrograms and summary of theta power change within different target regions and the NI soma area. The data for soma activation and inhibition in this Figure are identical to those presented in Figs. 2 and 3 for comparison purpose. Error bars (**c**, **e**, **g**, **j**, **l**, **n**) indicate SEM. *P *<* 0.05, **P *<* 0.01, ***P < 0.001, ****P *<* 0.0001; ns, not significant; Dunnett’s multiple comparisons test; see Supplemental Table 1 for detailed statistical analysis. Source data are provided as a Source Data file.

Mapping the axonal projection patterns of NI NMB neurons led us to examine the role(s) of the downstream target brain areas of NI NMB neurons. We stimulated ChR2-expressing axonal terminals from NI NMB neurons in four major targets and silenced the cell bodies by infusing the GABA receptor blocker muscimol into the NI to minimize antidromic activation^48^ (Fig. 7a). Stimulating axonal terminals in the MS and IPN significantly promoted locomotion speed, pupil-linked arousal level, and hippocampal theta power, with the MS particularly impactful on locomotion, and the IPN being more impactful on arousal (Fig. 7b–g). Interestingly, such stimulation in the LH significantly increased pupil size but did not change locomotion or theta power (Fig. 7b–g), despite the fact that many NI NMB neurons send axonal collaterals to target these brain areas (Supplementary Fig. 11). These results provide anatomical supports for our findings that multiple projections of NI NMB neurons are able to regulate locomotion, arousal, and hippocampal theta, each of which processes might be controlled by overlapping but different projection targets.

Because the results from optogenetic stimulation might be compounded by axonal collaterals and interconnections between downstream target brain areas, we carried out optogenetic inhibition to further examine the contribution(s) of specific projections. We expressed GtACR1 in NI NMB neurons and optogenetically inhibited GtACR1-expressing axonal terminals in various target areas (Fig. 7h). Inhibiting the axon terminals in the MS mildly but significantly reduced locomotor speed, dramatically reduced arousal, and had essentially no effect on theta rhythms (Fig. 7i–n). In contrast, inhibiting the projections to the IPN and the LH did not clearly change locomotion, arousal, or hippocampal theta (Fig. 7i–n). These results suggest that the projections from NI NMB neurons to the MS plays a prominent role in regulating arousal. While this pathway contributes to maintaining locomotion to a certain level, the control of locomotion and hippocampal theta by NI NMB neurons likely requires coordinated activity across their projections to multiple downstream targets.

Given the prominent role of NI projection to the MS, we directly activated MS neurons to probe the capacity of various MS cell types to promote locomotion, arousal, and hippocampal theta. The MS contains three major neuronal types: glutamatergic, GABAergic, and cholinergic^12,19,41,42^. We expressed ChR2 separately in these neuron populations by infusion Cre-dependent AAV vectors into the MS of *Vglut2-ires-Cre*, *Vgat-ires-Cre*, or *Chat-Cre* mice (Supplementary Fig. 12a). We analyzed the behavioral effects in response to stimulation at 10 and 50 Hz. Activating MS glutamatergic neurons significantly increased locomotor speed, arousal, and theta power (Supplementary Fig. 12b–k). Similarly, activating MS cholinergic neurons promoted locomotion, increased theta power, and enhanced arousal levels, especially when at the 50 Hz stimulation (Supplementary Fig. 12b–k). Consistent with previous studies^41,49^, activating MS GABAergic neurons during rest slightly induced theta rhythms, significantly increased arousal level, and essentially did not induce obvious locomotion (Supplementary Fig. 12b–k), suggested differential regulation of arousal and locomotion by MS GABAergic neurons. Together, these results indicate that MS neurons promote locomotion, arousal, and hippocampal theta in a cell type-specific manner.

## Discussion

Comparison of fiber photometry data, behavior assays, pupil-linked arousal assessment, and LFP recordings revealed correlations between NI NMB neuron activity and, respectively, locomotor speed, arousal level, and hippocampal theta power. We followed up on these observations by using cell-type-specific optogenetic activation and inactivation of NI NMB neurons to demonstrate functional roles of NI NMB neuron activity for each of these three processes. Subsequently, we conducted detailed virus-based circuit mapping studies and electrophysiological recordings, and thusly confirmed the existence of monosynaptic and reciprocal projections between NI NMB neurons and a variety of limbic and brainstem regions associated with these processes. Finally, projection-specific optogenetic mapping revealed projection-specific roles of NI NMB neurons in behavioral control.

Our findings indicate that NMB neurons in the NI, an understudied structure in the central gray area of the pons, concomitantly control locomotor speed, arousal, and hippocampal theta rhythms. Since these processes are often linked during locomotion^12–14^, one could reasonably argue that the NI is important for only one of these processes, for example exploratory locomotion that adaptively modulates other processes (e.g., pupil dynamics and/or hippocampal theta rhythms). However, multiple lines of experimental evidence argue against this potential confounding interpretation. First, blocking locomotion with pancuronium does not disrupt the capability of stimulating NI NMB neurons to heighten arousal and facilitate hippocampal theta oscillations. Although this does rule out the effect of motor commands as pancuronium blocks the final stage of the effector mechanisms, our results suggest that the effect of NI activation on arousal and hippocampal theta are not secondary to locomotion; similarly, reducing arousal with clonidine does not reduce the stimulating effect of these neurons on locomotion and theta power, suggesting that the effect of NI activation on locomotion and hippocampal theta are not secondary to arousal (Fig. 4). Second, we show that NI NMB neurons are activated during causal induction of arousal (with airpuff stimulus), even in the absence of locomotion (Supplementary Fig. 3b). This type of separate regulation has also been previously demonstrated: Vinck et al.^14^ showed that locomotion offset is followed by a slow decrease in pupil diameter that did not reach baseline values until fully 40 s later, thus illustrating a substantial period of elevated arousal in the absence of locomotion^14^. Third, we found that locomotion, hippocampal theta, and arousal levels could be differentially manipulated by activating specific projections of NI NMB neurons or specific cell types in the MS. Finally, electrical stimulation of the NI has been demonstrated to induce hippocampal theta rhythms in anesthetized animals, which obviously lack locomotion or any arousal responses^18^.

The NI has been traditionally considered as a periventricular area for stress responses because it receives functional inputs from corticotropin-releasing factor-expressing neurons^24–26,50^. Indeed, a substantial number of NI NMB neurons express CRF receptors (Supplementary Fig. 1). Previous work has reported that very high expression levels of *c-Fos* mRNA can be induced in the NI in response to several neurogenic stressors, including forced swimming^24,28^, continuous running on a treadmill for 60 min^51^, and footshock^52^. Note that each of these stressors either comprises locomotion or immediately elicits locomotion in the case of footshock. Here, using fiber photometry, we demonstrate that footshock but not quinine stress strongly activates NI NMB neurons, so it is clear that locomotion is an essential aspect of this activation. Moreover, we show that NI NMB neurons are also functionally involved in appetitive chasing. Although it is known that neurons in the periventricular nucleus—a well-established stress center—guide the formation of conditioned place aversion^53^, we found that stimulating NI NMB neurons lack such effect. Our results therefore support that NI NMB neurons only indirectly mediate stress responses.

Our findings substantially expand the earlier findings which provided initial clues that the NI may participate in integrative modulation of locomotion, arousal, and/or hippocampal theta. It was known that stimulating (or even ablating) the NI promotes locomotor activity^16,17^. Moreover, non-cell-type-specific chemogenetic stimulation of the neurons within and near the NI promotes the cortical desynchronization that is often associated with increased arousal^15^. Similarly, LFPs in the NI were found to exhibit a synchronized oscillatory theta pattern during hippocampal theta periods in anaesthetized rats^54,55^. However, technological challenges have long complicated the detailed characterization of the functional roles of specific neuron types and specific projections of the NI. Recall that the NI is surrounded by the LDT, the raphe nuclei, and the LC, the MLR, each of which has been implicated in locomotor control, arousal, and/or hippocampal theta oscillation^5,7,27,56,57^. Thus, electric stimulation that is not precisely targeted to NI neurons could easily trigger off-target activations of closely adjacent non-NI neurons or even fibers of passage. This is further complicated by the fact that stimulating neurons outside of the locomotor circuit, such as strongly activating pain-responsive neurons through tail pinches, can trigger rapid locomotion. Here we generated the NMB-Cre mouse line that allowed us to examine the role of NI neurons in controlling the behavioral and physiological processes of locomotion, arousal, and hippocampal theta oscillation. In addition to optogenetic stimulations in the NI and its various downstream stations, we performed fiber photometry and optogenetic inhibition to establish necessity and sufficiency of NI NMB neuronal activity for coordinating locomotor speed, pupil-linked arousal level, and theta power during both exploratory and appetitive navigations.

Combining mono-transsynaptic retrograde tracing and cell type-specific anterograde tracing revealed that NI NMB neurons receive inputs from several subcortical areas, such as the MS, LH, LHb, IPN, DRN, MRN, that are associated with arousal, locomotion, theta rhythms, and motivation^6,12,36,40,41,58–60^. These neurons likely integrate convergent inputs regarding animal motivational states and in turn project to the IPN, LH, and MS, brain areas known to function in regulating locomotion, arousal, and hippocampal theta^6,12,36,41,42^. Optogenetic stimulation of axonal terminals from NI NMB neurons to the IPN or the MS strongly promotes locomotion, arousal, and hippocampal theta, whereas activating those to the LH has a stimulatory effect only on arousal. The area-specific responses to terminal stimulations might reflect the functions of different downstream targets of NI NMB neurons, whereas collateral activation might contribute to some of the common effects for the terminal stimulations. Acutely inhibiting these neurons within the NI made animals come to a full stop abruptly, reduced arousal levels, and desynchronized hippocampal LFP. Terminal inhibitions show that only inhibiting the projection to the MS effectively reduces arousal and slightly decelerate locomotion. These results suggest that projections from NI NMB neurons to the MS regulate arousal and multiple downstream brain regions of NI NMB neurons together mediate the effects on locomotion and hippocampal theta.

The MS receives both GABAergic and glutamatergic innervations from the NI neurons^21,50^, including NMB neurons as shown here, and consists of glutamatergic, GABAergic, and cholinergic neurons. A recent study reveals that NI GABAergic neurons in mice reduces animal locomotion, reduces hippocampal theta, and control fear memory by targeting both glutamatergic and cholinergic neurons in the MS and sending collateral projections to somatostatin-positive interneurons in the hippocampus, thus make mice move slower and theta oscillations weaker^50^. Here we find that NI NMB neurons promotes locomotion, arousal, and hippocampal theta by targeting the MS and several other brain areas. This cell population represents a subset of NI neurons and includes both GABAergic (~3/4) and glutamatergic (~1/4) types. Our observation that NI glutamate neurons produces stronger effects than GABA cells does not necessarily conclude that mainly glutamate mediates the stimulatory effects of NMB neurons, because only a subset of NI GABA neurons express NMB. Resembling NMB neurons in the NI, Pro-opiomelanocortin (POMC) neurons in the arcuate nucleus represent a mixture of GABA cells and glutamate neurons^61^. Moreover, the arcuate nucleus has many GABA cells that are non-POMC cells, such as Agouti-related peptide neurons that have the opposite functions of POMC neurons^62^. It is thus possible that different neuron populations within the NI synergistically regulate various behavioral processes by acting at specific cell types in distinct downstream brain areas. Dissecting the functionally relevant GABAergic and glutamatergic NMB outputs in a cell type- and brain area-specific manner will be an exciting topic to pursue in the future.

Collectively, our findings establish major roles for the NI NMB neurons in controlling locomotor speed, arousal, and theta rhythms through their projections to multiple downstream brain areas. The ability to coordinately regulate locomotor speed, arousal level, and hippocampal theta power processes would offer an animal an added degree of flexibility for their actions, which would improve an animals’ chance of survival in a dynamic environment, as in navigating at a fast speed during predation or evasion.

## Methods

### Animals

All procedures were conducted with the approval of the Animal Care and Use Committee of the National Institute of Biological Sciences, Beijing, in accordance with governmental regulations of China. All mice were maintained on a 12 h reverse light/dark cycle (light on 8 PM) and given *ad libitum* access to chow and water except for the food-chasing task. *NMB-Cre* heterozygous mice were maintained on a mixed FVB/N & C57BL/6J background. Adult (8-16 weeks old) *Vgat-ires-Cre* mice [STOCK B6J.129S6(FVB)-*Slc32a1*^*tm2(cre)Lowl*^/MwarJ, NO: 028862] and *Vglut2-ires-Cre* mice [STOCK Slc17a6tm2(cre)Lowl/J, NO: 016963] of were obtained from the Jackson Laboratory (USA). The *Chat-Cre* transgenic mice [Tg (*ChAT-Cre*)24Gsat] were provided by MMRRC (Davis, CA, USA). Wildtype C57BL6/N mice were purchased from VitalRiver (Beijing, China). Mice were at least six weeks old at the time of surgery. Unless stated otherwise, all studies employed a mixture of male and female mice, and no differences between sexes were observed. All procedures were conducted during the dark cycle.

### Generation of *NMB-Cre* Mice

To generate *NMB-Cre* Mice, the bicistronic *NMB-Cre* allele was constructed by homologous recombination at the endogenous *NMB* locus, aided by targeted CRISPR endonuclease activity^63^. Briefly, homologous regions and the sequence encoding *Cre* were captured into a plasmid using a recombineering approach. The *Cre* sequence was inserted just after the endogenous START codon. The final targeting vector contained 2.0 kb homologous arms and was verified by both restriction digestion and sequencing. To generate site-specific double stranded breaks using CRISPR, a sgRNA sequence was selected such that the guide sequence would be separated from the PAM site in the genomic DNA by the Cre insertion. This ensured that the targeting vector and recombined *NMB* allele were protected from Cas9 nuclease activity. The Cas9-sgRNA target site (CCGGCGTCGCTCCCTTCAAC) was followed by the PAM sequence TGG in the exon 1 of the NMB-encoding gene. Super-ovulated female FVB/N mice were mated to FVB/N stud males, and fertilized zygotes were collected from oviducts. Cas9 protein, sgRNA, and targeting vector DNA were mixed and injected into the pronucleus of fertilized zygotes at the Model Animal Research Center of Nanjing University. Injected zygotes were implanted into oviducts of pseudopregnant CD1 female mice. Out of 103 pups genotyped, four were positive for the *NMB-Cre* knockin. The transgene was bred out by backcrossing to C57Bl/6J mice. All *NMB-Cre* mice used here were heterozygotes (genotype maintained in a mixed FVB/N & C57Bl/6J background). Founder pups and offspring were genotyped for the presence of the knockin allele by PCR.

### Virus production and surgery

Supplementary Table 3 shows the viral vectors used in this study. AAV vectors carrying the *DIO-ChR2-mCherry, DIO-mCherry, DIO-GCaMP6m*, *DIO-GFP* or *DIO-GtACR1-P2A-GFP* were packaged into serotype 2/9 vectors with titers ~2 × 10^12^ particles/ml. The *pAAV-EF1a-DIO-hChR2(H134R)-mCherry* construct was a gift from Dr. Karl Deisseroth (Addgene plasmid #20297). We constructed these plasmids by replacing the coding region of *ChR2-mCherry* in the *pAAV-EF1a-DIO-ChR2-mCherry* plasmid with that of *GCaMP6m* (Addgene Plasmid #40754), *EmGFP* (Addgene Plasmid #14757), or *GtACR1-P2A-GFP*. The sequence of *Guillardia theta* Anion Channel Rhodopsins 1 (*GtACR1*) was synthesized according to the original report^64^.

For targeted viral delivery, we performed stereotaxic injections using standard stereotaxic instruments (RWD Instruments, China) under Avertin anesthesia (i.p. 250 mg kg^−1^). A small craniotomy was made and a calibrated pulled-glass pipette (Sutter Instrument) was lowered to the NI (coordinates −5.4 mm from Lambda, 0 mm from the midline, and 3.6 mm ventral to Lambda). We infused 100–300 nL of virus solution (speed at 46 nL min^−1^) using a microsyringe pump (Nanoliter 2000 Injector with the Micro4 controller, WPI). We left the injection pipette in place for five additional minutes before withdrawing it slowly.

For optical manipulation, following virus injection mice were implanted with custom-built fiber connectors (fiber: 0.39 numerical aperture or NA, 200 μm diameter; Thorlabs). The tip of the fiber was lowered to 100 μm above the injection site in the NI. Implants were fixed to the skull with Cyanoacrylate adhesive (TONSAN 1454) and dental cement. For head-fixed preparations, individual mice were implanted with a custom-made titanium head-plate. Virus was allowed to express for 2 weeks before experiments. At the end of the behavioral analyses, we sacrificed the subject mice, performed standard histology, and confirmed the efficiency of both AAV infection and fiber placement. For delivery of sucrose and quinine, an intra-oral cheek fistula was implanted in mice following a previously described procedure^65^.

### Fiber photometry recording and electrophysiology

To record the NI neural activity, locomotion, arousal, and theta rhythms, we used a conditioning reward-retrieval task in which an odor cue (1% saturated n-amyl acetate vapor) was presented for 0.5 s, followed by a 5 s response time window. More than 3 s of running time during this time window resulted in delivery of a 5% sucrose solution reward for 1 s (i.e., 10 μL). The inter-trial interval (ITI) durations were randomly set in a range between 30 and 50 s. The timing for stimulus delivery was controlled through an USB-6008 DAQ (National Instrument) using a custom-developed LabVIEW program. Mice were allowed to habituate on a wheel treadmill in 2 daily sessions (about 30 min per session) prior to recordings. Before training, the mice were water-deprived for 36 h. Each mouse underwent 2–5 daily training sessions (30-50 trials/day) to achieve a stable performance (defined as >90% correct).

After training, we conducted recordings from the NI and the hippocampal CA1 region of head-fixed mice during the performance of locomotion training task on the same wheel treadmill. On the initial recording day, a small craniotomy was made over CA1 (−2.0 mm anterior posterior, 1.5 mm lateral, relative to Bregma) under light isoflurane anesthesia and then the mouse was allowed to recover for 2 h. Mice were then fitted with a head-plate and secured on the wheel treadmill before electrodes were lowered. A tungsten microelectrode recorded CA1 LFPs. Electrodes were initially lowered to ~1.4 mm. All LFP recordings were referenced to the surface of the olfactory bulb. A fiber photometry system was used to record GCaMP6 fluorescence change from a mouse on a wheel treadmill. An infrared camera was used to measure pupil diameter, which was used to monitor arousal. A rotary encoder was used to monitor locomotion. The drinking nozzle was placed near the tongue of the head-fixed mouse and an infrared laser beam was used to detect lick signals. Signals were digitized and recorded by a customized OpenEphys board (http://www.open-ephys.org/). LFP data were filtered through a low-pass filter (200 Hz cut-off). All data were sampled at 1 kHz.

For experiments in which airpuff were used to arouse the mouse, a small tube was positioned behind the head, based the method of Vinck et al^14^. Airpuff were delivered to the body of the mouse with minimum waiting intervals of 60–80 s with pseudorandom timing. Airpuff were generated by a nitrogen tank using a solenoid-operated piston pump (SMC pneumatics) to control timing and amplitude. The intensity of the airpuff was adjusted during each experiment such that a noticeable increase in arousal—as indexed by pupil diameter—could be observed, without resulting in locomotion. For the airpuff analyses, we only included trials that occurred during quiescence epochs, and had a maximum of 2 cm movement across the analyzed interval.

### Cue-associated delivery of footshock, quinine, and sucrose

Individual GCaMP6m-expressing *NMB-Cre* mice was placed in an acrylic box (25 × 25 × 30, *L* × *W* × *H* in cm) with a metal grid floor that delivered footshock currents (0.6 mA scrambled, 0.5 s). We delivered 30 trials that coupled an auditory cue (8 kHz, sine wave, 70 dB, 2 s) to the delayed presentation (2 s) of a footshock (0.5 s; ITI 20–40 s). For intra-oral infusion of sucrose or quinine, a peristaltic pump (AniLab) was used to infuse 10 μL of either quinine (5 mM) or sucrose (5% w/v) through Silastic tubing into the oral cavity (speed 20 μL s^−1^). An auditory tone (4 kHz for quinine and 12 kHz for sucrose, sine wave, 70 dB, 2 s) was presented for 2 s followed by 2 s delay and then 0.5 s of sucrose or quinine infusion (ITI 20–40 s for sucrose and ITI 110–130 s for quinine). Note that water was withheld (36 h) from the post-surgery mice that underwent treatments involving sucrose.

### Optogenetic manipulation

The blue laser power (473 nm, 10–20 mW) at the end of the patch cable was verified at the start of the experiment. Laser pulses were delivered only after a mouse stayed quiescent within a 5 s period. Laser stimulation (5 ms per pulse at the frequencies of 5/10/20/50 Hz) or inhibition were delivered randomly in 50% of the trials (at least 20 trials) with inter-trial interval (ITI) durations at 30–50 s. For optogenetic inhibition, laser (5 s) were delivered only after a mouse continuously walked for 5 s. The laser pulse duration and frequency were controlled by a MASTER8 controller (A.M.P.I., Israel). The timing for stimulus delivery was controlled through an USB-6008 DAQ (National Instrument) using a custom-developed LabView program. Mice were allowed to habituate on a wheel treadmill in 2 daily sessions (about 30 min per session) prior to recordings. On the initial test day, a small craniotomy was made over CA1 (see above). LFP, pupil diameter, and locomotion were monitored as described above.

For stimulation of NI NMB projections in different target regions, 150 nL muscimol (0.7 mM in ACSF) was applied to the NI via an injection pipette ten mins before behavioral tests to silence the NI neurons. To decouple locomotion from arousal, we injected the adrenergic alpha-2 receptor agonist clonidine hydrochloride (0.1 mg kg^−1^, ip; 4205-91-8, Sigma-Aldrich) to reduce arousal 10 min before behavioral tests. To decouple theta oscillation from locomotion, we applied the muscle relaxant pancuronium bromide (0.18 mg kg^−1^, ip; P1918-10MG, Sigma-Aldrich) to suppress locomotion 10 min before behavioral tests.

### Food-chasing task

Mice were food-restricted to maintain 90% of a healthy body weight^66^. Individual mice with an implanted optical fiber were then placed in an open field chamber (54 × 72 × 30, L × W × H in cm) in which food pellets were available only on a food tray. During the habituation phase (day 0), mice were allowed to reach for multiple pellets (14 mg, Bio-serv) in the food tray held in a single, stationary location. During the following days (day 1–5), the food tray holding the pellets moved when the mouse walked to within a 20 cm distance of the tray. We controlled the movement of the food tray by embedding a magnet within the food tray and moved another magnet underneath the training chamber via motorized 2D sliders. An overhead camera monitored animal body positions (10 frames s^−1^). Upon the detection of a mouse walking close to the food tray (distance < 20 cm), a custom-written LabVIEW program triggered the movement of motorized sliders; the food tray was moved at a speed of 20 cm s^−1^. The food tray paused for 1.5 s for food retrieval after a mouse sustained chasing behavior for 5 s. The daily session ended after a mouse retrieved a maximum of 15 pellets within 15 min. For optogenetic inhibition on day 3, blue light was delivered continuously to the NI of GtACR1-expressing *NMB-Cre* mice while the food tray was moving. We defined an attempt as the act of triggering food tray movement. We only classified a given chasing attempt as a success when the mouse collected at least one food pellet after it had initiated chasing. The mean speed of the daily session was calculated by averaging the speed of the body movement following the initiation of chasing attempts.

To record NI NMB neural activity in the food-chasing task, GCaMP6m-expressing *NMB-Cre* mice were habituated on day 0; on day 1, mice were then tested with the task while a fiber photometry system was used to record the Ca^2+^ signal from NI NMB neurons.

### Conditioned place preference (CPP)

We performed the CPP test as our previously described procedure^58^. The entire CPP test consisted of three phases: the preconditioning phase on day 1, the conditioning phase on days 2 and 3, and the test phase on day 4. During the preconditioning phase, individual mice were allowed to freely explore the entire apparatus for 15 min. During the conditioning phase, mice were first confined to one side chamber for 20 min. Trains of blue light pulses (50 Hz, 5 ms, 5 s on/10 s off) were passed into the NI of ChR2- or mCherry-expressing mice. Continuous blue light was delivered (5 s on/10 s off) to the NI of GtACR1-expressing mice. Twenty-four hours later, the mice were confined to the opposite side for 20 min with an implanted optical fiber, but no light was delivered. During the test phase, the mice were allowed to freely explore the entire apparatus for 15 min.

### Real-time place preference test

We placed individual mice in a custom-made behavioral arena (50 × 50 × 30 cm white plastic chamber) for 15 min per session. The wall between the left and the right chamber was half-open for the freely behaving of mouse connected with optical patch cable. We conducted baseline and stimulation sessions, and examined the effect of stimulation or inhibition of NI NMB neurons. In the activation experiments, animals that entered the optogenetic-stimulation chamber received blue laser pulses (5 ms per pulse at the frequencies of 50 Hz, 20 mW, 470 nm). We used 5 s on/5 s off laser stimulation protocol to avoid continuous running so that the mouse cannot cross back into the non-stimulation side, that might cause false-positive reward phenotype. In the inhibition experiments, the mice received constant blue laser (4 mW, 470 nm) until they crossed back into the non-stimulation side. The locations of the mouse were assessed from the video recording data using a custom MATLAB program.

### Open field test

Mice were individually placed in a 30 × 30 × 30 cm white plastic chamber in well-lit room and allowed to move freely for 30 min. Animals’ locomotion was recorded with an overhead camera. Total travel distance traveled and time spent in the 15 × 15 cm center of the square were quantified.

### Quantitative real-time PCR

For quantification of NMB transcript levels, wildtype, heterozygotes, and homozygotes of NMB-Cre mice were deeply anesthetized by Avertin (i.p.) and decapitated, and their brains were extracted. Olfactory bulb and the NI region were manually dissected in cold ACSF for RNA extraction. We performed RT-PCR using 5× All-In-One RT MasterMix (Applied Biological Materials, cat# G590) according to the instructions. We performed qPCR using 2 × RealStar Green Fast Mixture (GenStar cat# A301-10) according to the instructions provided in the kit. Quantitative qPCR was performed in triplicates for each condition. NMB qPCR primers: Fwd: TCCCTTCAACTGGGATCTCCCG and Rev: GCTGGGCCACTGAAGTTCAT.

### Plasma epinephrine measurements

We performed optogenetic stimulation on an open field arena (45 × 45 × 30 cm). Trains of blue light pulses (5 ms per pulse at the frequencies of 50 Hz; 5 s on/10 s off; 10 min) were delivered through the optical fiber to the NI of ChR2-expressing mice or mCherry-expressing control mice with the same transgenic background. Blood samples were collected 20 min after stimulation of the NI. Five drops of blood were drawn from the retrobulbar intraorbital capillary plexus and centrifuged at 1000*g* for 15 min at 4 °C. Supernatant was collected and plasma epinephrine concentrations were measured using commercially-available ELISA kits (Abnova cat# KA1882).

### Electrophysiological recordings in brain slices

We performed electrophysiological recordings as our previously described procedure^58^. To characterize neurotransmitters released by the NI NMB neurons toward the three different target structures (the MS, the LH, or the IPN). A 300 nL volume of *AAV9-EF1a-DIO-ChR2-mCherry* was injected into the NI of *NMB-Cre* mice. To confirm that the NI NMB neurons were monosynaptically connected to the four selected upstream regions (the MS, the LH, the LHb, or the IPN), a 300 nL volume of *AAV9-EF1a-DIO-mGFP* was injected into the NI of *NMB-Cre* mice and a 300 nL mixture of *AAV9-hSyn-Cre* and *AAV9-EF1a-DIO-ChR2-mCherry* (equal volumes) was injected to the upstream region.

After virus expression for about 2 weeks, coronal sections (200 μm thick) were prepared. To label recorded cells, Neurobiotin (0.25% w/v) was added to the internal solution. Pharmacological experiments used 10 μM, DNQX (AMPA receptor antagonist) and 10 μM Gabazine (GABA_A_ receptor antagonist). To test for monosynaptic connections, we performed pharmacological experiments in the ACSF solution containing 1 μM of the sodium channel blocker TTX and 100 μM of the potassium channel blocker 4-AP. Synaptic release was evoked by flashing 473 nm blue light (5 ms pulse, 1 Hz, 30 s interval). EPSCs and IPSCs were measured while holding the membrane potential at −65 mV and −10 mV, respectively. After the recordings, brain slices were fixed in 4% PFA in 0.01 M PBS. The slices were then incubated with blocking buffer containing 0.3% Triton X-100 and 3% bovine serum albumin (BSA) in PBS for 1 h at room temperature. To visualize Neurobiotin-filled cells, slices were incubated with Alexa Fluor cy2-conjugated streptavidin (1:500, ThermoFisher Scientific). Images were acquired using a Zeiss LSM510 Meta confocal microscope.

### Immunohistochemistry and in situ hybridization

Mice were killed with an overdose of pentobarbital and perfused intracardially with 0.1 M phosphate buffer saline and then 4% paraformaldehyde. After cryoprotection in 30% sucrose, coronal sections (40 µm thickness) were cut on a cryostat (Leica CM1950). After rising with 0.3% Triton-X in 0.1 M PBS (PBST) and blocking with 3% (w/v) normal bovine serum (BSA) in PBST for 1 h, the brain sections were incubated with primary antibodies in the blocking solutions at 4 °C for 48 h (anti-Rln3, AF3107, 1:40, R&D Systems; anti-CRFR1, OAEB02329, 1:500, Aviva System Biology). After rising, the brain sections were incubated with the secondary antibody (Alexa 488-conjugated donkey anti-goat for Rln3; biotin-conjugated donkey anti-goat for CRFR1) for 2 h at room temperature. To label CRFR1, the brains sections were incubated with avidin-biotinylated horseradish peroxidase complex (PK-6100; 1:50; Vector Laboratories) diluted in 3% BSA for 0.5 h, and then followed by incubation in tyramide-488 (B40953; 1:100; Thermo Fisher Scientific) diluted in 3% BSA for 0.5 h.

To characterize the specificity of Cre expression in the NI of *NMB-Cre* mice. *AAV9 -DIO-mCherry* was injected to the NI of *NMB-Cre* mice. Following virus expression, we perfused the mice and performed in situ hybridization (NMB or VGAT probe) using RNAscope® 2.5 HD Detection kits (Advanced Cell Diagnostics) according to the instructions provided in the kit. For characterization of fiber placements and tracing experiments, images were imaged with an automated slide scanner (VS120 Virtual Slide, Olympus). Other images were imaged using a Zeiss LSM510 Meta or Nikon A1 confocal microscope. We analyzed and quantified images by using FIJI (https://imagej.net/Welcome).

### Data analysis

#### Analysis of GCaMP signal and locomotion

We exported the photometry data from Spike2 or OpenEphys to MATLAB as mat files for further analysis. We first smoothed data with the MATLAB *smooth* function. The values for fluorescence change (*ΔF/F*) were defined as (*F* − *F*_0_)/*F*_0_, where *F*_0_ is the baseline fluorescence signal averaged over a 2.5 s-long control time window, which was typically set 0.5–3 s preceding the trigger events. GCaMP signals and locomotor speed signals were further smoothed by binning (50- or 100-ms bins) for presentation purposes. The locomotion speed was extracted from the output of the rotary encoder. We resampled the locomotion signal to 10 Hz and smoothed. To calculate the GCaMP signals during the resting and locomotion stages, we considered a mouse movement of <1 cm s^−1^ as resting and movement >2 cm s^−1^ as locomotion, and calculated the mean *ΔF/F* value for both the resting and the locomotion stages. The Pearson correlation coefficients between fluorescence change values and locomotor speed data were computed using the MATLAB *corrcoef* function. The transitions between rest and walking were defined as the change between a rest period (<0.1 cm s^−1^ for at least 3 s) and an active period (>1 cm s^−1^ for 3 s and peak speed >7 cm s^−1^). We used multivariate permutation tests to analyze the statistical significance of the event-related fluorescence (ERF) change. We used 5000 permutations for the α-level of 0.01 to compare the *Δ*F/F values of all trials at each time point against the mean of ERF baseline values (0.5–3 s before event onset). A series of P-value at each time point were generated and the results were superimposed on the average ERF curve with red lines indicating statistically significant (*P* < 0.01) increase.

#### Measurement of pupil diameter

Pupil diameter data was extracted from gray-scale video frames of 640 × 320 pixels at 20 fps. We used a similar method as that described by Vinck et al^14^. Briefly, we set programmatically outlined eyes and then set a threshold based on the median intensity value for the whole image, to remove background pixels. After thresholding, fuzzy c-means clustering was used to identify two clusters of pixels, a process that typically yielded one cluster of relatively dark pixels for the pupil and a second cluster of relatively light pixels for the rest of the eye. We then fit an ellipse to the detected edges using least-squares fitting and determined pupil diameter as the major axis length of the ellipse. Normalized pupil diameter was defined as the pupil diameter at each point divided by the mean of the baseline (0.5–3 s before event onset). To align pupil diameter signal to the trial onset, we used a LED cue in the video.

#### Analysis of hippocampal LFPs

All field potential spectra were computed with the MATLAB *spectrogram* function with a 1 s time window at a resolution of ∆*F* = 0.1 Hz and ∆*T* = 20 ms. The theta rhythms before and after activation and inhibition of the NI NMB neurons was quantified by peak theta frequency and normalized theta power. Theta (6–10 Hz) power is normalized to the power between 0.1 and 10 Hz. Normalized theta power change was defined as the mean theta power at every point divided by the mean of the baseline (0.5–3 s before event onset). To calculate correlation matrices, the GCaMP signal, locomotion speed, normalized pupil diameter, normalized theta power, and lick signal data were resampled 10 points per s.

#### Analysis of in vitro electrophysiology data

The physiological data were analyzed using Clampfit 10 software (Molecular Devices). To quantify EPSCs or IPSCs amplitudes, five to eight sweeps of PSCs were averaged for each data point. The amplitudes of EPSCs or IPSCs were measured by subtracting the peak with the mean of the baseline immediately before the stimulation. The effects of DNQX on EPSCs and of Gabazine on IPSCs were compared by calculating amplitude changes before and ~7 min after drug application. Latency is defined as time between stimulus onset and 10% of IPSC or EPSC amplitude. All amplitude data for EPSCs and IPSCs is presented in Supplementary Table 2.

#### Quantification of anterograde and retrograde tracing

We used Imaris (Bitplane) to quantify GFP-positive pixels or presynaptic cells. Regions of interest (ROI) were manually outlined according to the Paxinos & Franklin Mouse Brain Atlas^67^. We counted the total number of GFP-positive pixels in each ROI. The count data were normalized between animals based on each animal’s NI fluorescence signals. The total number of presynaptic cells in each upstream brain region was divided by the total number of trans-synaptically labeled cells.

#### Statistical analysis

All data are presented as mean ± SEM. Inferential statistical tests were carried out using GraphPad Prism (version 6.01) software or MATLAB (R2016a), and all tests were two-tailed. *P < 0.05; **P < 0.01; ***P < 0.001; ****P < 0.0001; ns, not significant for all statistical analyses presented in figures. Details about the statistical analyses are reported in Supplementary Table 1.

### Reporting summary

Further information on research design is available in the Nature Research Reporting Summary linked to this article.

## Supplementary information

Supplementary Information

Reporting Summary

Description of Additional Supplementary Files

Supplementary Movie 1

Supplementary Movie 2

Supplementary Movie 3

Supplementary Movie 4

## Data Availability

The data that support the findings of this study are available from the corresponding author upon reasonable request. The source data underlying Figs. 2e, g, l, m, 3f, h, j, 4e, f, g, 5g, i, k, m, 6f, h, j, 7c, e, g, j, l, n and Supplementary Figs. 1d–f, 2e, j, k, 4f, g, j–l, 5c, f, g, h, 6g, h, 7a–c, 8j, 9h, 10c, e, h, i, 12d, g, j, k are provided as a Source Data file.

## Source data

Source Data

## Code availability

The code used in this study are available from the corresponding author upon reasonable request.
