# Supplementary material for: Control of locomotor speed, arousal, and hippocampal theta rhythms by the nucleus incertus
Source: Nat Commun. 2020 Jan 14;11:262. doi: 10.1038/s41467-019-14116-y (PMC6959274; doi:10.1038/s41467-019-14116-y)
Supplement: Supplementary file 1 — Supplementary Information [file 41467_2019_14116_MOESM1_ESM.pdf]

Control of Locomotor Speed, Arousal, and Hippocampal Theta Rhythms by the Nucleus Incertus

Lu et al.

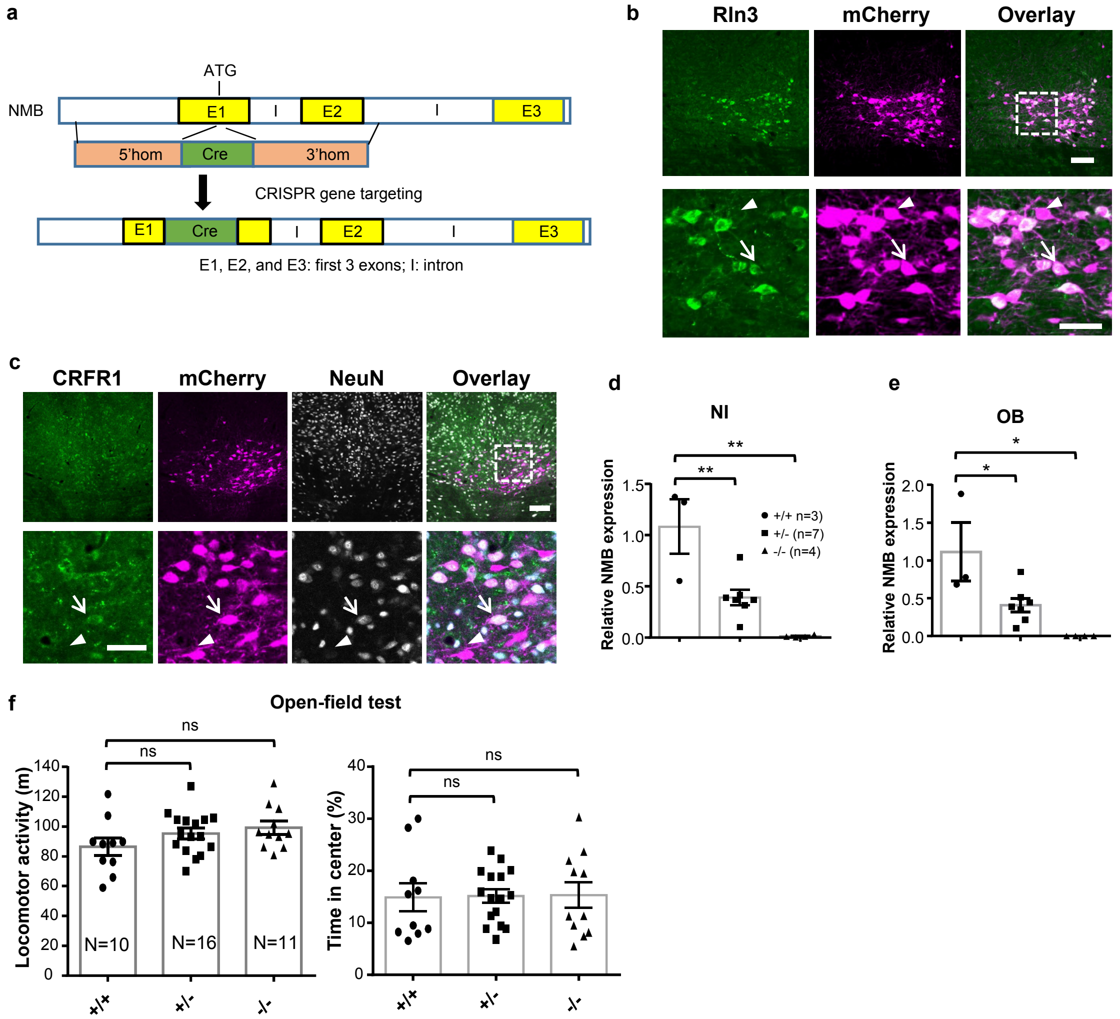

Supplementary Figure 1 | Characterization of NMB-Cre mice. (**a**) Schematics showing the targeting vector and knock-in strategy for generating *NMB-Cre* mice. The Cre-recombinase was inserted after the start codon at the endogenous NMB allele. E1, E2, and E3 indicate the first three exons and I indicates intron. (**b**) Up, the presence of Relaxin-3 (Rln3, green) in some mCherry-expressing (red) NI NMB neurons (~50%, 518 overlay/1026 mCherry$^+$ neurons; ~82%, 518 overlay/630 Rln3$^+$ neurons; 15 sections from 3 mice); Bottom, the zoom-in view of the dashed rectangular area in the right up image. Arrows, dual-labeled cells; Arrowheads, mCherry+ cells that clearly lacked Rln3 expression. (**c**) The presence of CRFR1 (green) in some mCherry-expressing (red) NI NMB neurons (~37%, 369 overlay/1006 mCherry$^+$ neurons; ~60%, 369 overlay/610 CRFR1$^+$ neurons; ~33%, 1006 mCherry$^+$ neurons /3053 NeuN$^+$ neurons; 14 sections from 2 mice). Bottom, the zoom-in view of the dashed rectangular area in the right up image. (**d, e**) qPCR of NMB transcripts in the NI and the olfactory bulb (OB) of wild type (+/+; n = 3), heterozygous (+/-; n = 7), and homozygous (-/-; n = 4) NMB-Cre mice. NMB transcript levels were normalized to actin transcript levels. (**f**) Heterozygous or homozygous NMB-Cre mice show normal basal locomotor activity or anxiety level as monitored by an open field test. Summary graphs depict the total length traveled in 30 min during an open field test (left), and the time the mice stayed in the center of the open field (right) as a function of different genotypes of NMB-Cre mice. Error bars (d-f) indicate SEM. Scale bar = 100 µm (b, c, up), 50 µm (b, c, bottom). *$P < 0.05$, **$P < 0.01$; ns, not significant; Unpaired t test; see Supplemental Table 1 for detailed statistical analysis. Source data are provided as a Source Data file.

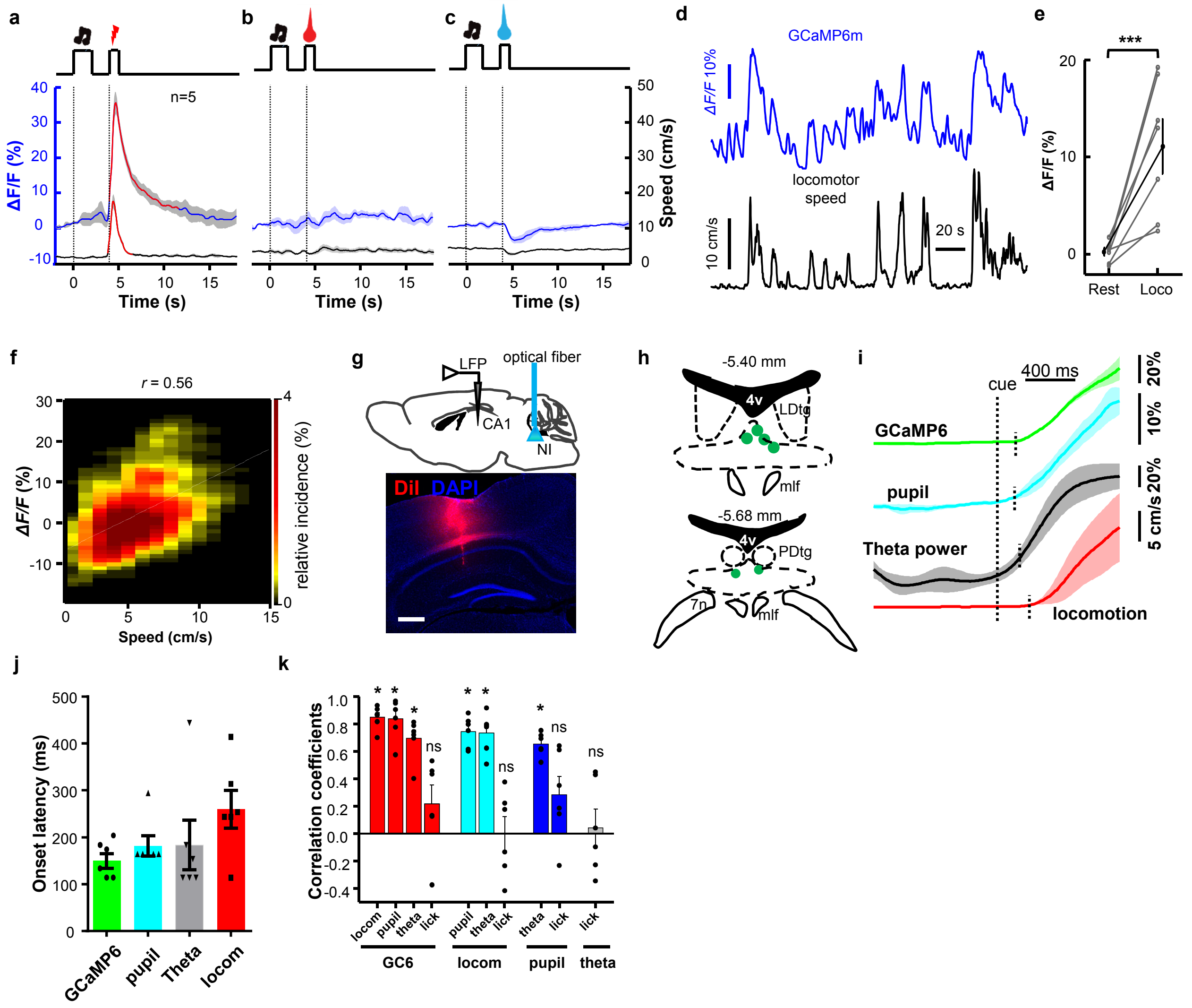

Supplementary Figure 2 | Correlation between the activity of NI NMB neurons and locomotion, arousal, and theta power. (**a**) GCaMP signals of NI NMB neurons in a cue-coupled footshock paradigm (n = 5 mice). Red segments indicate statistically significant increase from the baseline (*P* < 0.01; multivariate permutation test). (**b**) No responses to intra-oral infusion of quinine while also no change of locomotor speed. (**c**) Mild inhibitory responses to intra-oral infusion of sucrose accompanied by locomotion decreasing. (**d**) Representative GCaMP signals of NI neurons and the locomotor speeds of a mouse engaged in exploratory locomotion in an open field. (**e**) GCaMP signals during locomotion is significant larger than that in rest (n = 7 mice). (**f**) Correlation between calcium signals and locomotion speeds for the entire group of GCaMP6-expressing mice tested in an open field (n = 7 mice). Relative incidence means the probability of the locomotor speed (binned in 1 cm s^-1) and the corresponding calcium signal on all recording data. (**g**) Schematic drawing of the simultaneous recording sites in the hippocampal CA1 and NI of NMB-Cre::DIO-GCaMP6 mice (up) and representative coronal sections showing DiI-labeled electrode track in the CA1 (bottom). Scale bar, 500 µm. (**h**) Postmortem identification of the tip positions of optical fibers in the NI (n = 6 GCaMP mice used in Figure 1). LDTg, laterodorsal tegmental nucleus; PDTg, posterodorsal tegmental nucleus; mlf, medial longitudinal fasciculus; 4v, 4th ventricle; 7n, facial nerve. Numbers above indicate the distance from Bregma. (**i, j**) Mean (i) and latency to onset (j) of GCaMP6 fluorescence, pupil diameter change, theta power change, and locomotor speed (n = 6 mice, same data as shown in Figure 1e). (**k**) Bar plot for the correlation coefficients between GCaMP6 fluorescence changes and various behavioral parameters (same data as shown in Figure 1f). Shaded areas (a-c, i) and error bars (e, j, k) indicate SEM. **P* < 0.05, ****P* < 0.005; ns, not significant; Wilcoxon rank sum test and one sample t test; see Supplemental Table 1 for detailed statistical analysis. Source data are provided as a Source Data file.

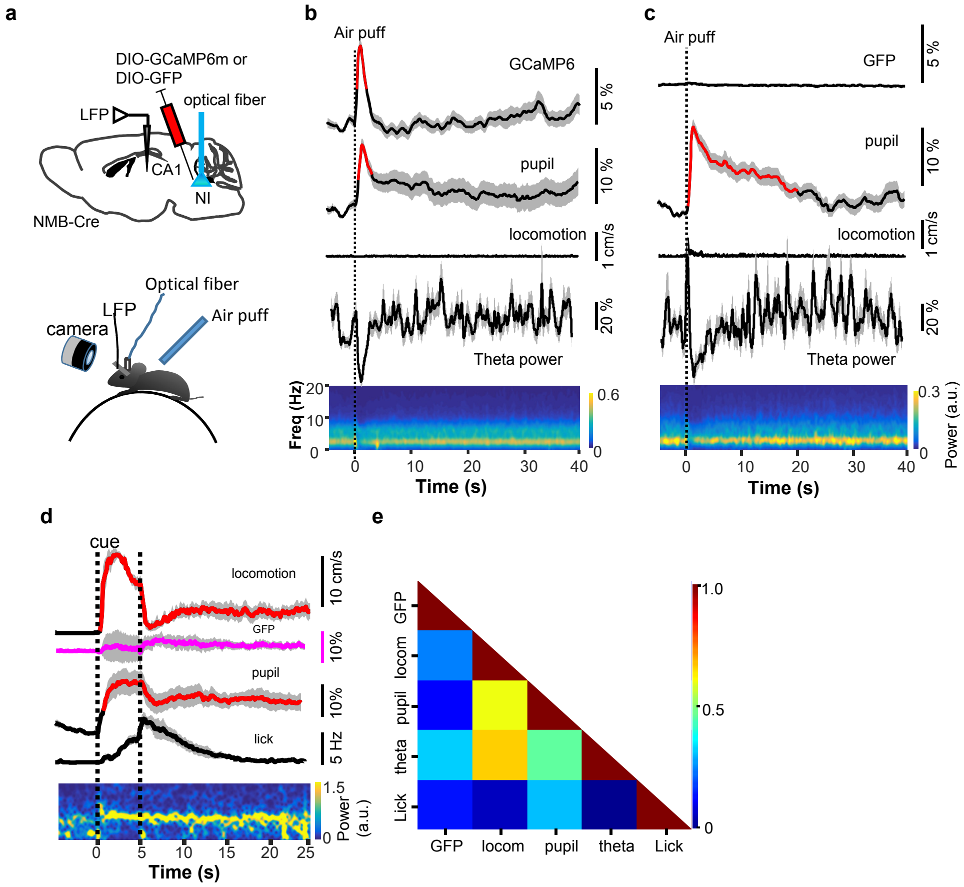

Supplementary Figure 3 | The activity of NI NMB neurons is correlated with arousal level. (**a**) Schematics showing the method of simultaneous fiber photometry from the NI, local field potential recording from the hippocampal CA1, and behavioral monitoring of NMB-Cre mice that received airpuff in their back. (**b**) Average GCaMP signals, pupil diameter, locomotor speed, and LFP spectrograms as a function of time around the airpuff (n = 156 trials in 11 mice). The correlation coefficient between GCaMP signal intensity and pupil diameter change is 0.66 and the peak GCaMP fluorescence increase is 7.1%. (**c**) No change in green fluorescence level during airpuff of control mice that expressed GFP in the NI NMB neurons (n = 75 trials in 5 mice). (**d**) No change in green fluorescence level during locomotion of control mice that expressed GFP in the NI neurons (n = 7 mice; control experiment for Figure 1c-1f). (**e**) Cross-correlation analysis of GFP fluorescence signal, locomotor speed, normalized pupil diameter, LFP theta band power change, and lick rates (control experiment for Figure 1f). Color scale to the right indicates correlation values. Shaded areas (b, c, d) indicate SEM.

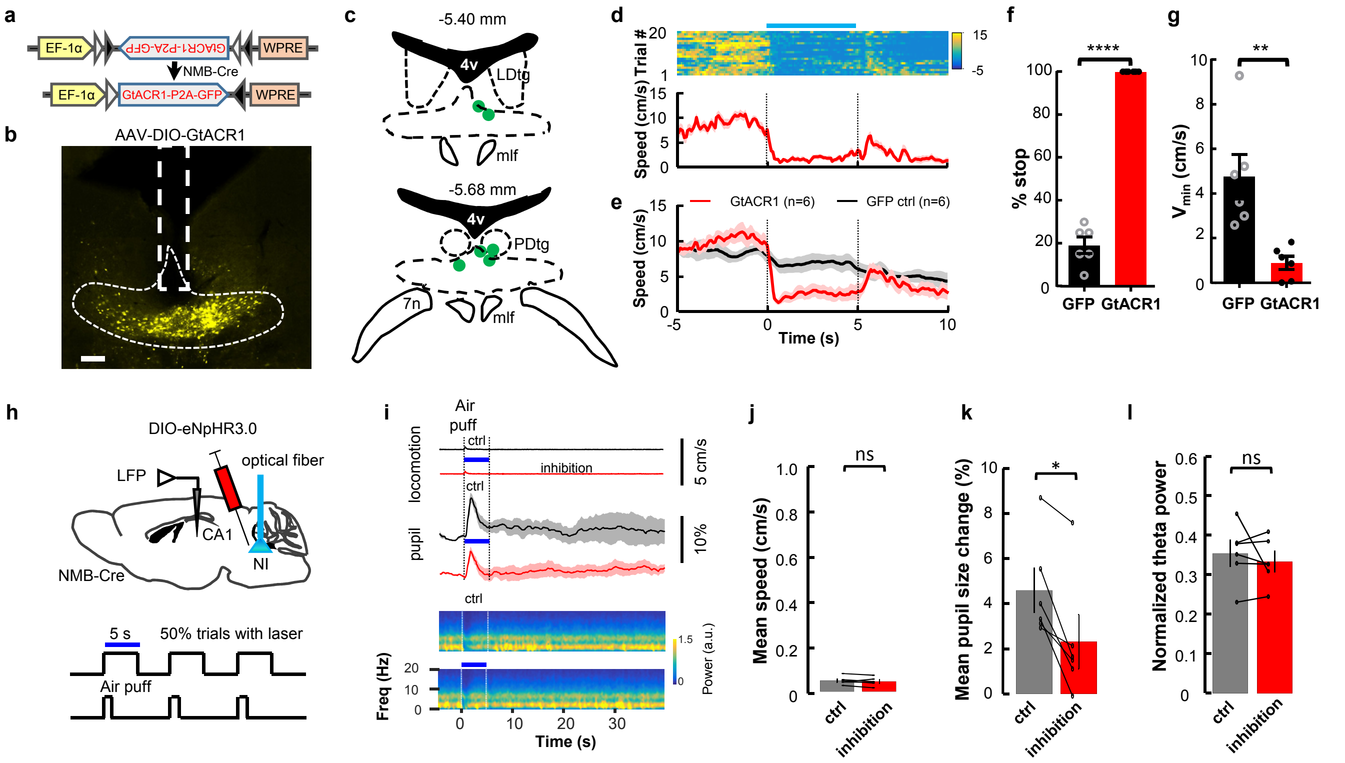

Supplementary Figure 4 | The activity of NI neurons is required for locomotion and arousal. (**a**) Schematic showing Cre-dependent bicistronic expression of GtACR1 and GFP in NI NMB neurons. (**b**) A coronal section showing the pattern of GFP-expression following the injection of *AAV-DIO-GtACR1-P2A-GFP* vectors into the NI of a *NMB-Cre* mouse. Scale bar = 200 µm. (**c**) Postmortem identification of the tip positions of optical fibers that were used to inhibit GtACR1-expressing NI neurons (n = 6 GtACR1 mice used in Figure 2). (**d**) Trial-by-trial heatmap representation of locomotor speed and the peri-event plot of the average speed for a mouse show that optogenetic inhibition of NI NMB neurons immediately aborted the locomotor activity of head-fixed GtACR1-expressing mice. (**e**) Locomotor speed aligned to laser onset. (**f**) Number of stops during laser inhibition for each group. (**g**) Minimum speed during laser inhibition. (**h**) Schematics showing the experiment of testing the effect of inhibiting NI NMB neurons in immobile mice aroused with airpuff. (**i**) Average locomotor speed, pupil diameter change traces, and grand average of LFP spectrograms of trials with (red lines) or without (black lines) optogenetic inhibition. Vertical dash lines indicate laser delivery (561 nm, 20 mW, 5 s continuous light). (**j-l**) Summary of locomotor speed (j, mean speed during inhibition, 0 to 5 s), pupil diameter change (k, mean pupil diameter change during inhibition), and theta power (l, theta power is normalized to the sum of 0.1 to 10 Hz power). Shaded areas (d, e, i) and error bars (f, g, j-l) indicate SEM. *$P < 0.05$, **$P < 0.01$, ****$P < 0.0001$, ns, not significant; Unpaired t test and paired t test; see Supplemental Table 1 for detailed statistical analysis. Source data are provided as a Source Data file.

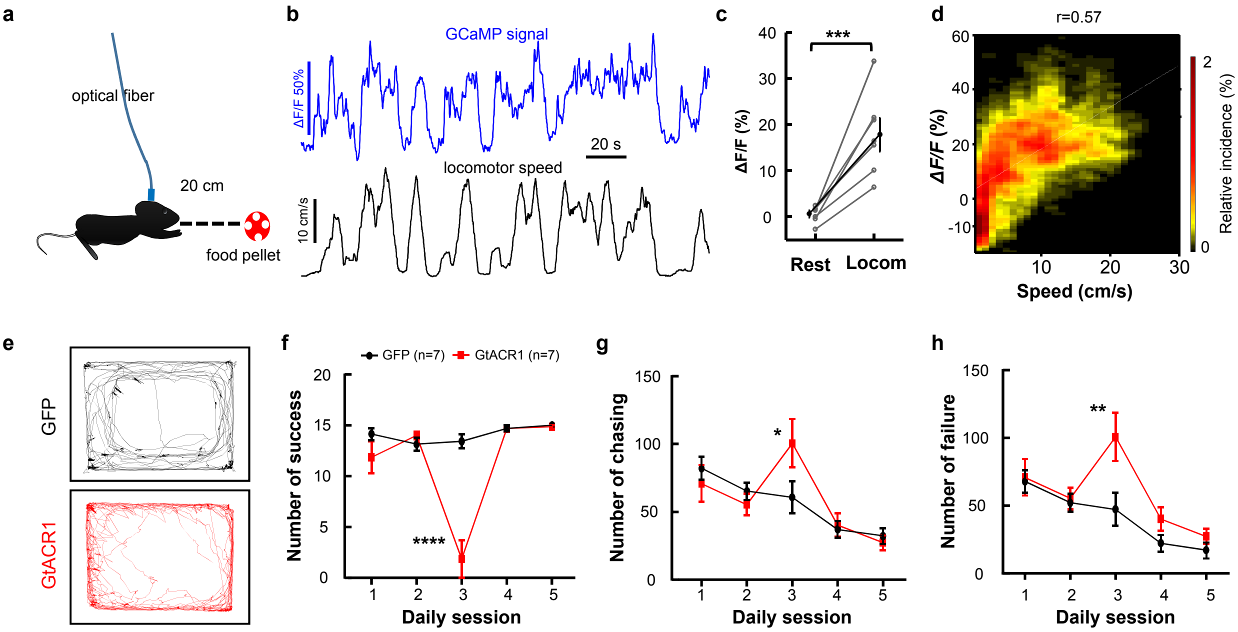

Supplementary Figure 5 | The activity of NI neurons is required for appetitive locomotion. (**a**) Schematic showing the method of recording GCaMP signals from a mouse engaged in appetitive locomotion. In the food-chasing task, a previously food-deprived mouse needed to chase after a moving food tray (20 cm·s-1) to retrieve a food pellet. (**b**) Raw traces showing the NI GCaMP signals and simultaneously recorded locomotor speed of a mouse. (**c**) GCaMP signals of NI NMB neurons during rest and active pursuit of the food pellets (n = 7 mice). (**d**) Correlation of GCaMP signal amplitudes and delay-adjusted locomotor speeds of the mouse shown in (b). Relative incidence means the probability of the locomotor speed (binned in 1 cm s^-1) and the corresponding calcium signal on all recording data. (**e**) Raw locomotion tracks of a control mouse and an experiment mouse within a food-chasing test session with optogenetic inhibition. (**f-h**) Optogenetic inhibition of NI neurons decreased the number of success (f) but increased the number of chasing attempts (g) and the number of failures in the food-chasing task (failure trials in control group: 47/61 chases; experiment group: 99/101 chases; h). Error bars (c, f-h) indicate SEM. **P* < 0.05, ***P* < 0.01, ****P* < 0.001, *****P* < 0.0001; Sidak's multiple comparisons test; see Supplemental Table 1 for detailed statistical analysis. Source data are provided as a Source Data file.

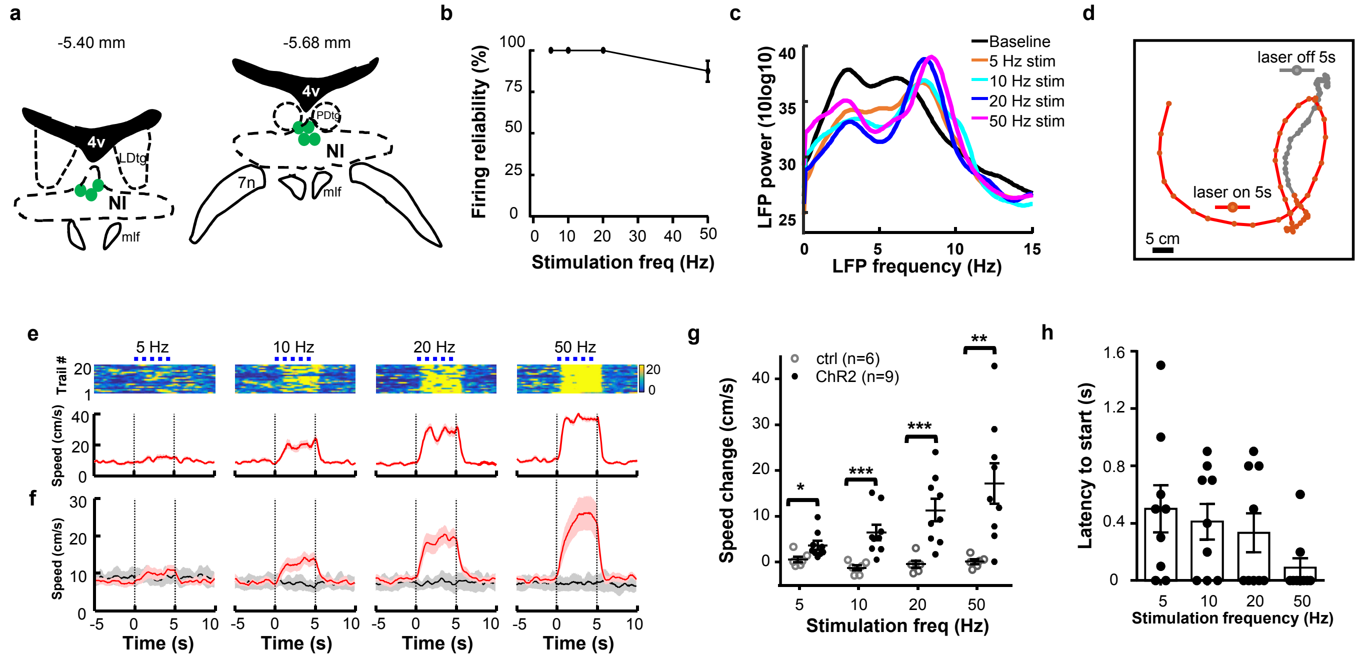

Supplementary Figure 6 | Activating NI NMB neurons increases locomotor activity in an open field arena. (**a**) Postmortem identification of the tip positions of optical fibers for optogenetic stimulation (n = 7 ChR2 mice used in Figure 3). (**b**) Mean ratio of successful spike generation in relationship to light stimulation at different frequencies (n = 4 cells). (**c**) The increase in theta power caused by activation of NI NMB neurons by different stimulation frequencies (n = 6 mice). (**d**) Example of altered motor activity during laser stimulation (50Hz, 5ms). Lines represent the mouse's path; dots represent the mouse's location every 0.1 s. Gray path, 5 s of activity before stimulation; red path, 5 s of activity during stimulation. (**e**) The effects of delivering light pulses at different frequencies (5 ms pulses for 5 s; 20-40 s inter-trial interval, 20 trials) on the locomotor speed of a mouse in an open field. Heatmaps draw locomotor speed of individual trials. (**f**) Locomotor speed aligned to different frequency laser onset or without stimulation (ChR2 in NI, red line, n = 9 mice; mCherry in NI, black line, n = 6 mice). (**g**) Summary of the stimulation effects on speed change (the mean speed 0-5s before laser on subtracted from the speed 5 s after onset of laser stimulation). (**h**) The latency from the onset of stimulation to the significant increase in locomotor speed. Shaded areas (e, f) and error bars (g, h) indicate SEM. **P* < 0.05, ***P* < 0.01, ****P* < 0.001; Mann Whitney test; see Supplemental Table 1 for detailed statistical analysis. Source data are provided as a Source Data file.

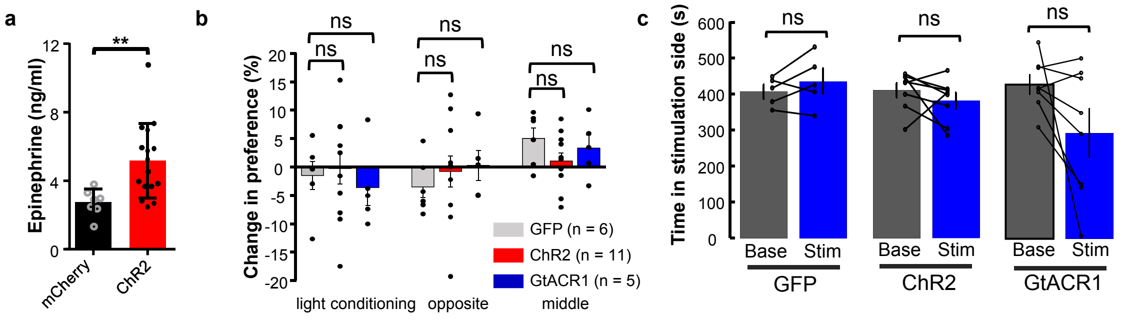

Supplementary Figure 7 | Activating NI NMB neurons increases plasma epinephrine level. (**a**) Optogenetic stimulation (50 Hz, 5 s on 10 s off, 10 min in an open field arena) of NI NMB neurons increased plasma level of epinephrine (n = 7 mCherry mice, 16 ChR2 mice). (**b**) Changes in the preference scores for the conditioned chamber, its opposite chamber, and the middle chamber following optogenetic activation or inhibition of NI neurons. (**c**) Bar plots showing that neither activating nor inhibiting produced significant real time place preference (n = 5 GFP mice, 8 ChR2 mice, 8 GtACR1 mice). Error bars indicate SEM. ***P* < 0.01; ns, not significant; Mann Whitney test and Tukey's multiple comparisons test; see Supplemental Table 1 for detailed statistical analysis. Source data are provided as a Source Data file.

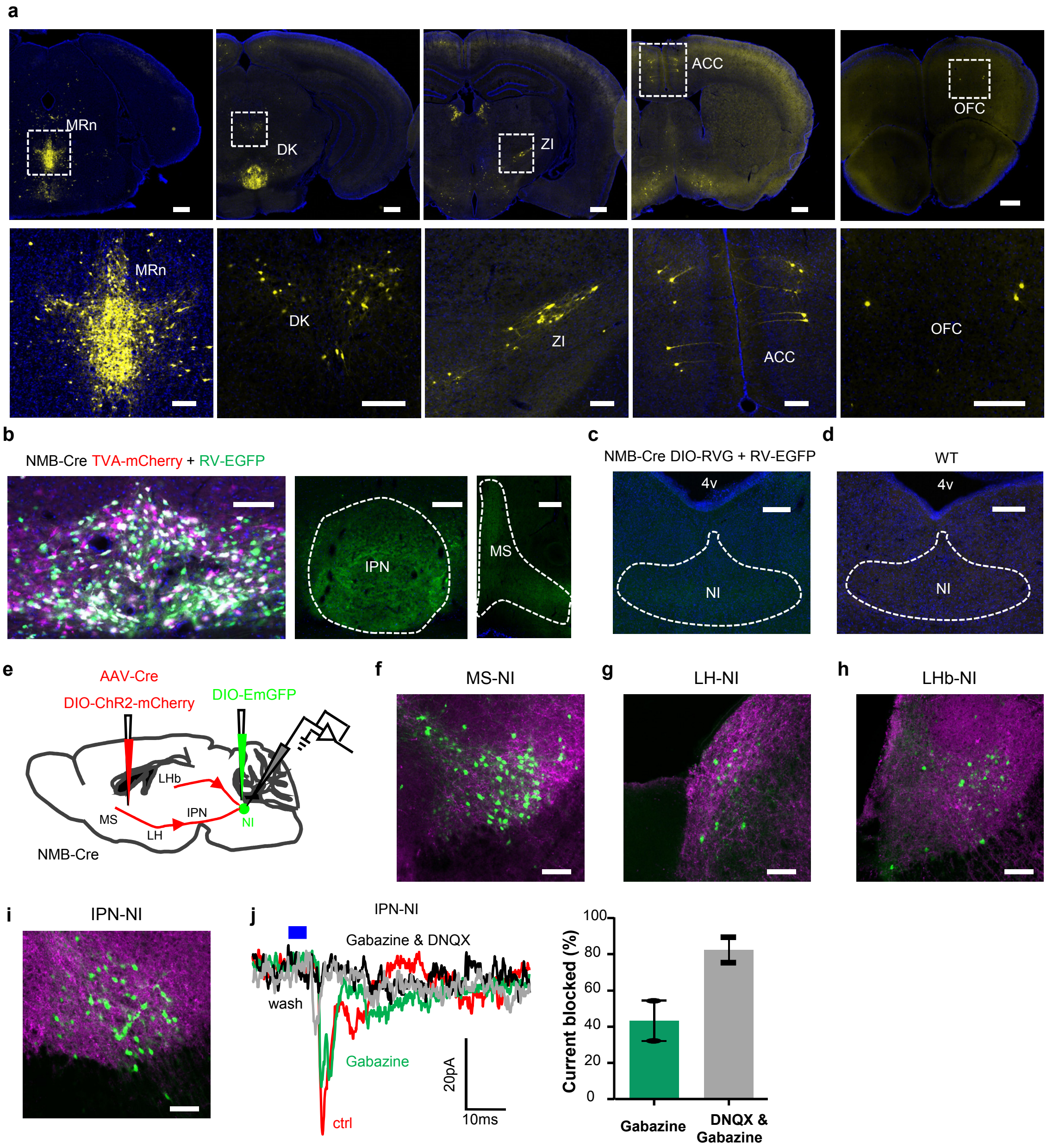

Supplementary Figure 8 | Characterization of presynaptic partners of NI NMB neurons. (**a**) Cell type-specific infection of recombinant rabies virus (RV) in NMB neurons led to transsynaptic labeling of input neurons. (**b**) Omission of AAV-G protein during injection resulted in absence of spread to presynaptic partners (middle and right), suggesting that the selective requirement of RV G protein expression for the initiation of transsynaptic virus spreading. (**c**) Omission of AAV-TVA during injection resulted in absence of RV-GFP expression, suggesting that the selective requirement of TVA expression for the infection of RV. (**d**) Injection AAV-DIO-G, AAV-DIO-TVA, and RV-GFP into the NI of a wildtype (WT) mouse resulted in absence of virus expression. (**e**) Schematic diagram showing the method of optogenetic stimulation of the ChR2-mCherry expressing axons from the MS, the LH, the LHb, or the IPN and recordings from the NI NMB^+ cells in brain slices. (**f-i**) Sagittal sections showing the expression of EmGFP (green) in the NI and ChR2-mCherry fibers (red) from the MS (f), the LH (g), the LHb (h), or the IPN (i). Anterior is to the left and dorsal to the top. 4v, 4th ventricle. (**j**) Representative recording traces from a NMB^+ neuron in the NI following photostimulation of $ChR2^+$ IPN neuron axonal terminals and summary of bath application of Gabazine and DNQX (latency, 9.2 ± 3.0 ms; n = 2 NMB^+ cells from 1 mice). Error bar (j) indicates SEM. Scale bar = 500 µm (a, up), 200 µm (a, bottom; c; d), 100 µm (b, f-i). Source data are provided as a Source Data file.

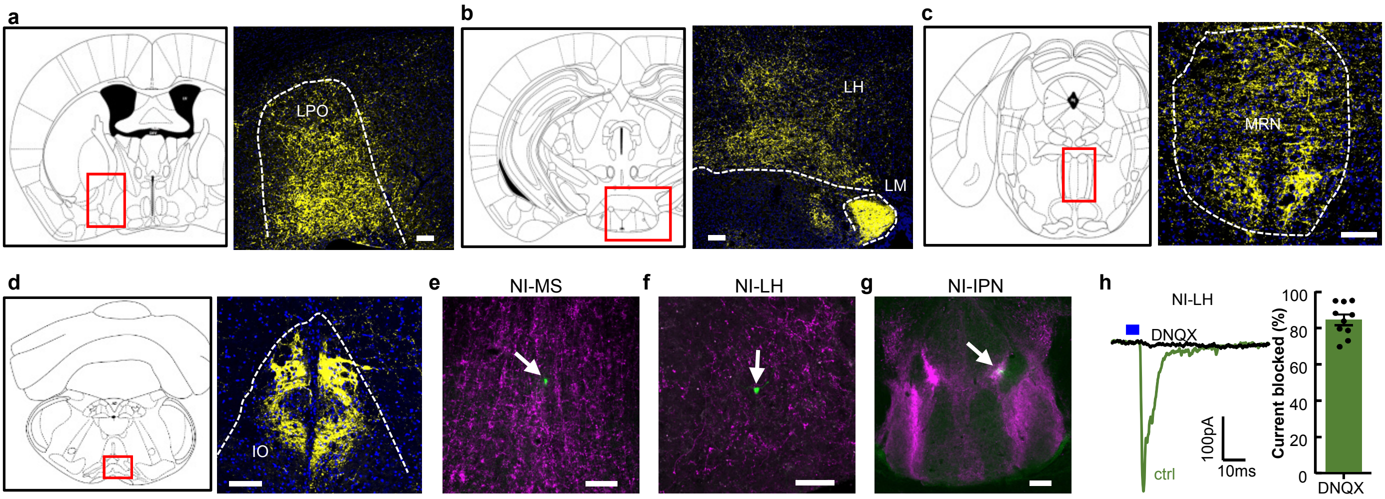

Supplementary Figure 9 | Characterization of axonal projection patterns of NI NMB neurons. (**a-d**) Infusion of *AAV-DIO-tdTomato-T2A-Synaptophysin-EGFP* into the NI of NMB-Cre mice resulted in the expression of synaptophysin-EGFP fusion protein (green) in the axonal projections from NI NMB neurons within the lateral preoptic area (LPO; a), the LH and the lateral mammary nucleus (LM; b), the medial raphe nucleus (MRN; c), and the inferior olive (IO; d). In all cases, the left panel is a stereotaxic map adapted from the Paxinos and Franklin mouse brain atlas, with the red box depicting the area for the image shown on the right panel; the right panel is a representative image of the boxed area showing the synaptic signal. (**e-g**) A cell in the MS (e), the LH (f) or the IPN (g) of a ChR2-expressing *NMB-Cre* mouse was filled with 0.5% Neurobiotin in the recording pipette and labeled with Cy2-streptavidin. Red indicates ChR2-mCherry^{+} fibers, and green indicates recorded cells. (**h**) Representative recording traces of light evoked EPSCs from a LH neuron and group data reveal that brief light stimulation of ChR2^{+} axonal terminals from the NI NMB neurons produced fast EPSCs that were blocked by DNQX (latency, 3.8 ± 0.5 ms; n = 10 cells from 6 mice). Error bar (h) indicate SEM. Scale bars = 100 μm (a-g). Source data are provided as a Source Data file.

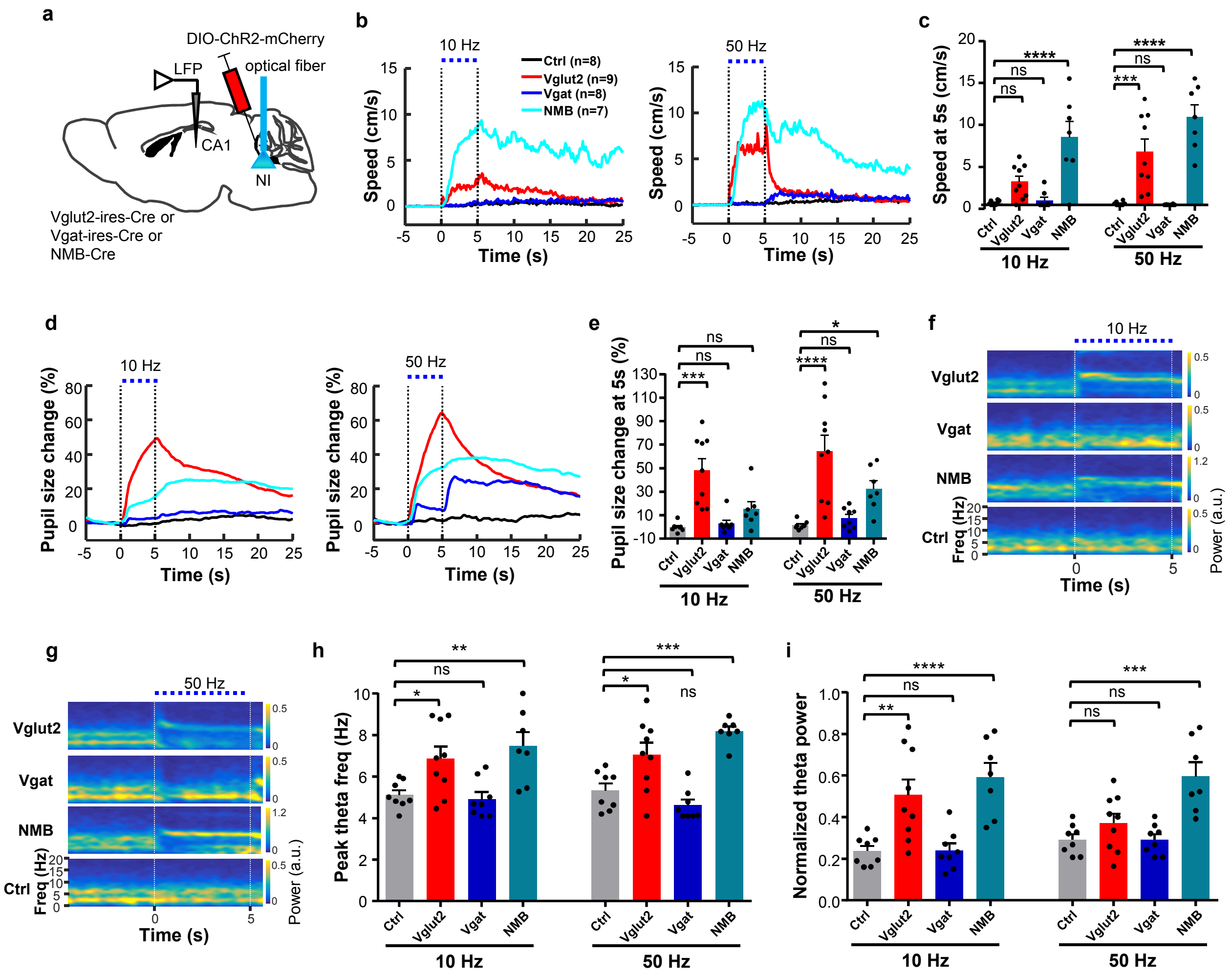

Supplementary Figure 10 | The effects of activating different NI cell types on locomotion, arousal, and theta power. (**a**) Experimental schematic for viral expression of ChR2 and stimulation of NI neurons. (**b**) Average locomotor speed traces for mice with glutamatergic (red, n = 9 mice), GABAergic (blue, n = 8 mice), or NMB+ (cyan, n = 7 mice) NI neurons stimulated (left, 10 Hz light; right, 50 Hz light). (**c**) Summary of locomotor speed elicited by stimulating different cell types in the NI. (**d**) Average pupil diameter change as a function of time around laser onset (left, 10 Hz light; right, 50 Hz light). (**e**) Summary of pupil diameter change elicited by stimulating different cell types in the NI. (**f, g**) Grand average of LFP spectrograms of optogenetic activation of NI neurons with 10 Hz (f) and 50 Hz (g) stimulation frequency. (**h, i**) Summary of peak theta frequency and theta (6 to 10 Hz) power change that elicited by stimulating different cell types in the NI (theta power is normalized to sum of 0.1 to 10 Hz power). All NMB data is same as corresponding stimulation frequency data in Figure 3 for comparison purpose. Error bars (c, e, h, i) indicate SEM. *P<0.05, **P<0.01, ***P<0.001, ****P<0.0001; ns, not significant; Unpaired t test.

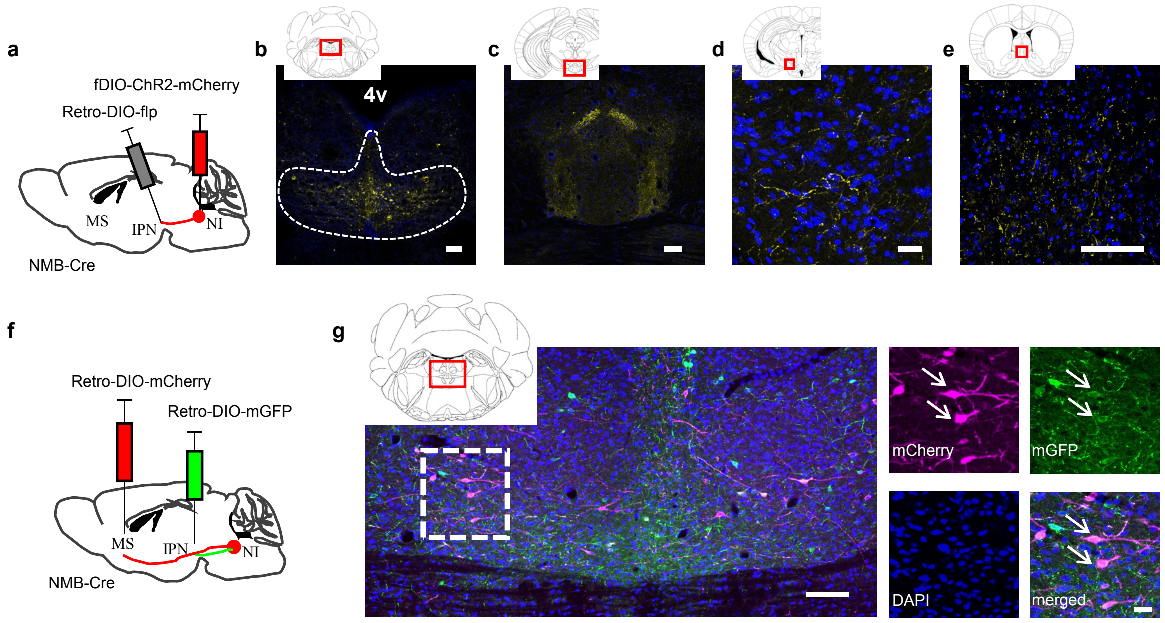

Supplementary Figure 11 | IPN-projecting NI NMB neurons send axon collaterals to the LH and the MS. (**a**) Specific labeling of NI NMB neurons projecting to IPN by using a two-step injection strategy. (**b-e**) Injecting AAV-retro-DIO-Flp into the IPN and AAV-fDIO-ChR2-mCherry into the NI of NMB-Cre mice results in the labeling of somata in the NI (b) and axon collaterals in the IPN (c), the LH (d) and the MS (e). (**f**) Schematic for labeling IPN- and MS-projecting NI NMB neurons. (**g**) Images show colocalization of IPN- and MS-projecting NI NMB neurons. Arrows, dual labeled NI NMB neurons. Scale bars = 100 µm (b; c; e; g, left), 25 µm (d; g, right).

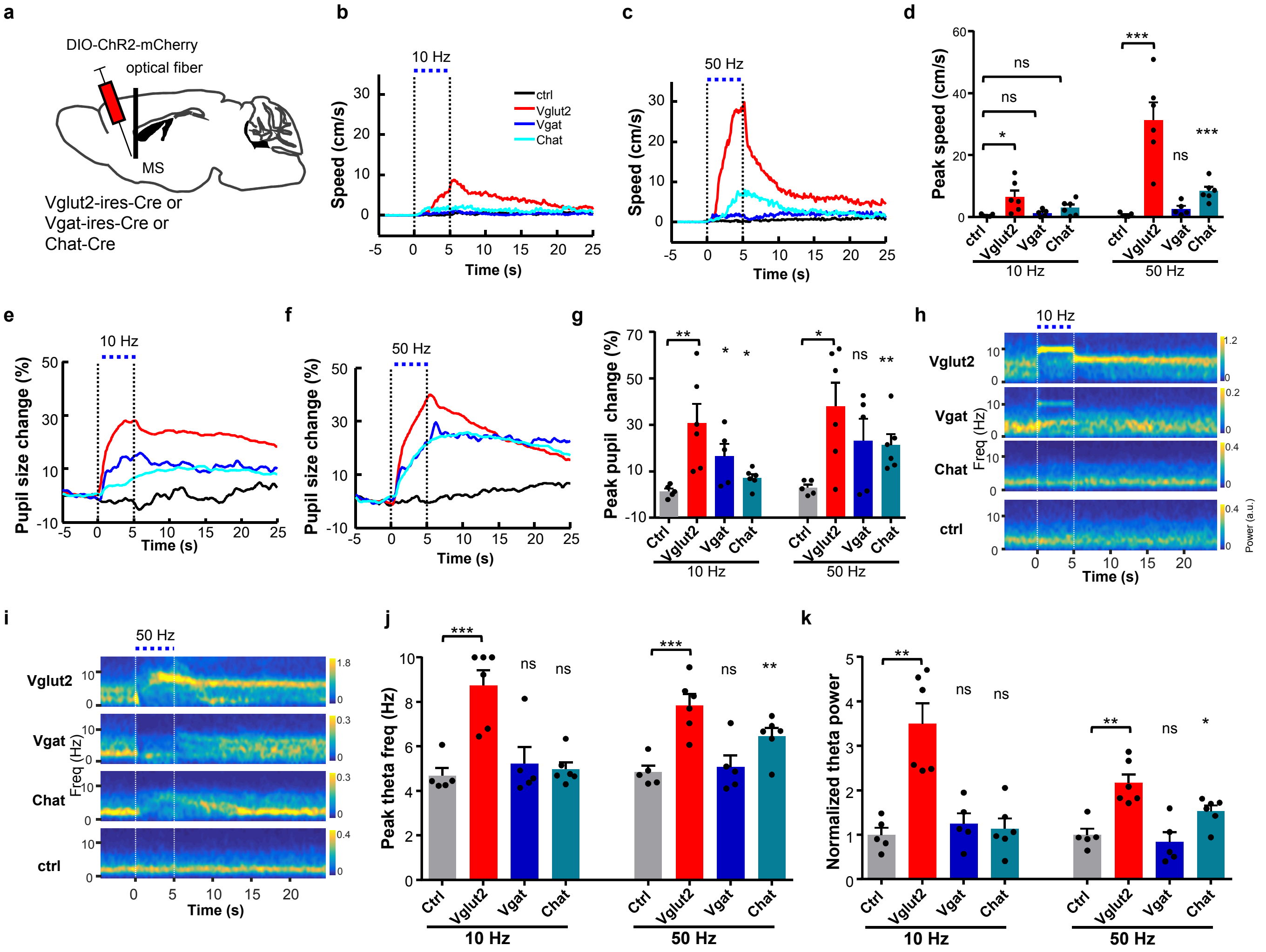

Supplementary Figure 12 | Activating MS glutamatergic and cholinergic neurons promotes locomotion, arousal, and theta power. (**a**) Experimental schematic for viral expression of ChR2 and stimulation of MS neurons. (**b, c**) Average locomotor speed traces following the stimulation of glutamatergic (red, n = 6), GABAergic (blue, n = 5), or cholinergic (cyan, n = 6) neurons in the MS. (**d**) Summary of the stimulation effects on locomotor speed. (**e, f**) Average pupil diameter as a function of time around laser onset. (**g**) Summary of the stimulation effects on pupil diameter. (**h, i**) Grand average of LFP spectrograms. (**j, k**) Summary of peak theta frequency (j) and theta power change (k) elicited by stimulating different cell types in the MS. Error bars (d, g, j, k) indicate SEM. **P* < 0.05, ***P* < 0.01, ****P* < 0.001; ns, not significant; Unpaired t test; see Supplemental Table 1 for detailed statistical analysis. Source data are provided as a Source Data file.

Supplementary Table 1: Summary of statistical analyses

Figure #	Conditions	n per group	Mean		SEM		Analysis	Factor	P value
2e	GFP vs GtACR1	6,6	14.7300	0.2010	2.2180	0.0937	Mann-Whitney test	inhibition	0.0022
2g	GFP vs GtACR1	6,6	-4.3300	-14.9000	0.9569	1.6210	Mann-Whitney test	inhibition	0.0022
2i	GFP vs GtACR1	6,6	0.7546	0.4986	0.0581	0.0741	Unpaired t test	inhibition	0.0216
2l	GFP vs GtACR1	7,7	13.1900	6.8150	1.1990	0.4891	Mann Whitney test	inhibition	0.0006
2m	GFP vs GtACR1	7,7	28.2800	1.1260	5.8980	1.1260	Unpaired t test	inhibition	0.0006
3f	GFP vs ChR2 5 Hz	6,7	0.7474	5.9240	0.3460	1.4540	Tukey's multiple comparisons test	stimulation	0.0293
	GFP vs ChR2 10 Hz	6,7	0.4717	8.0550	0.1995	1.8710	Tukey's multiple comparisons test	stimulation	0.0001
	GFP vs ChR2 20 Hz	6,7	0.1641	11.5500	0.0686	1.2470	Tukey's multiple comparisons test	stimulation	< 0.0001
	GFP vs ChR2 50 Hz	6,7	0.3164	10.4700	0.2159	1.4630	Tukey's multiple comparisons test	stimulation	< 0.0001
3h	GFP vs ChR2 5 Hz	6,7	4.7000	15.8000	3.2550	5.9170	Wilcoxon rank sum test	stimulation	0.0513
	GFP vs ChR2 10 Hz	6,7	-2.9300	15.1000	2.3380	6.3340	Wilcoxon rank sum test	stimulation	0.0140
	GFP vs ChR2 20 Hz	6,7	-6.3100	20.5000	1.5250	5.0920	Tukey's multiple comparisons test	stimulation	0.0008
	GFP vs ChR2 50 Hz	6,7	-3.1200	32.4000	1.6930	6.9960	Tukey's multiple comparisons test	stimulation	< 0.0001
3j	GFP vs ChR2 5 Hz	6,7	0.2301	0.5415	0.0229	0.0522	Tukey's multiple comparisons test	stimulation	0.0023
	GFP vs ChR2 10 Hz	6,7	0.2639	0.5927	0.0149	0.0696	Tukey's multiple comparisons test	stimulation	0.0011
	GFP vs ChR2 20 Hz	6,7	0.3015	0.6723	0.0263	0.0658	Tukey's multiple comparisons test	stimulation	0.0002
	GFP vs ChR2 50 Hz	6,7	0.2661	0.5964	0.0271	0.0668	Tukey's multiple comparisons test	stimulation	0.0010
4e	saline unstim vs stim	7,7	0.1699	6.5160	0.0353	0.6808	Paired t test	stimulation	< 0.0001
	clonidine unstim vs stim	7,7	0.1838	6.4880	0.0942	1.5950	Paired t test	stimulation	0.0086
	pancuronium unstim vs stim	4,4	0.3117	1.2780	0.1947	0.7232	Paired t test	stimulation	0.3156
	saline vs clonidine	7,7	6.5160	6.4880	0.6808	1.5950	Mann-Whitney test	clonidine	0.8718
	saline vs pancuronium	7,4	6.5160	1.2780	0.6808	0.7232	Mann-Whitney test	pancuronium	0.0121
4f	saline unstim vs stim	7,7	101.6000	144.7000	0.9358	8.5470	Paired t test	stimulation	0.0016
	clonidine unstim vs stim	7,7	101.6000	102.0000	0.6163	0.5950	Paired t test	stimulation	0.4907
	pancuronium unstim vs stim	4,4	102.4000	159.3000	0.9337	27.3800	Paired t test	stimulation	0.1221
	saline vs clonidine	7,7	144.7000	102.0000	8.5470	0.5950	Mann-Whitney test	clonidine	0.0006
	saline vs pancuronium	7,4	144.7000	159.3000	8.5470	27.3800	Mann-Whitney test	pancuronium	0.9152
4g	saline unstim vs stim	7,7	0.9864	2.0210	0.1649	0.1612	Paired t test	stimulation	< 0.0001
	clonidine unstim vs stim	7,7	0.9959	2.3620	0.0965	0.3354	Paired t test	stimulation	0.0072
	pancuronium unstim vs stim	4,4	1.0100	1.6410	0.0168	0.1085	Paired t test	stimulation	0.0069
	saline vs clonidine	7,7	2.0210	2.3620	0.1612	0.3354	Mann-Whitney test	clonidine	0.6031
	saline vs pancuronium	7,4	2.0210	1.6410	0.1612	0.1085	Mann-Whitney test	pancuronium	0.2182
7c	ctrl vs NI-MS ChR2 50 Hz	7,6	0.3911	8.5030	0.1493	1.2020	Dunnett's multiple comparisons test	stimulation	0.0002
	ctrl vs NI-LH ChR2 50 Hz	7,7	0.3911	3.5020	0.1493	0.7458	Dunnett's multiple comparisons test	stimulation	0.2392
	ctrl vs NI-IPN ChR2 50 Hz	7,5	0.3911	6.7490	0.1493	3.0950	Dunnett's multiple comparisons test	stimulation	0.0062
	ctrl vs NI-IO ChR2 50 Hz	7,6	0.3911	1.3740	0.1493	0.3791	Dunnett's multiple comparisons test	stimulation	0.9693
	NI vs NI-MS ChR2 50 Hz	7,6	12.9700	8.5030	1.0340	1.2020	Dunnett's multiple comparisons test	stimulation	0.0572
	NI vs NI-LH ChR2 50 Hz	7,7	12.9700	3.5020	1.0340	0.7458	Dunnett's multiple comparisons test	stimulation	< 0.0001
	NI vs NI-IPN ChR2 50 Hz	7,5	12.9700	6.7490	1.0340	3.0950	Dunnett's multiple comparisons test	stimulation	0.0075
7e	ctrl vs NI-MS ChR2 50 Hz	7,6	1.7000	15.4000	0.6061	5.3470	Mann-Whitney test	stimulation	0.0140
	ctrl vs NI-LH ChR2 50 Hz	7,7	1.7000	16.8000	0.6061	3.5450	Mann-Whitney test	stimulation	0.0006
	ctrl vs NI-IPN ChR2 50 Hz	7,5	1.7000	38.3000	0.6061	4.1830	Dunnett's multiple comparisons test	stimulation	< 0.0001
	ctrl vs NI-IO ChR2 50 Hz	7,6	1.7000	9.1000	0.6061	2.5780	Dunnett's multiple comparisons test	stimulation	0.6367
	NI vs NI-MS ChR2 50 Hz	7,6	32.7000	15.4000	6.9580	5.3470	Dunnett's multiple comparisons test	stimulation	0.0367
	NI vs NI-LH ChR2 50 Hz	7,7	32.7000	16.8000	6.9580	3.5450	Dunnett's multiple comparisons test	stimulation	0.0478
	NI vs NI-IPN ChR2 50 Hz	7,5	32.7000	38.3000	6.9580	4.1830	Dunnett's multiple comparisons test	stimulation	0.8559
7g	ctrl vs NI-MS ChR2 50 Hz	7,6	0.9815	1.6480	0.0944	0.1785	Dunnett's multiple comparisons test	stimulation	0.0189
	ctrl vs NI-LH ChR2 50 Hz	7,7	0.9815	1.3540	0.0944	0.1960	Dunnett's multiple comparisons test	stimulation	0.3782
	ctrl vs NI-IPN ChR2 50 Hz	7,5	0.9815	1.8670	0.0944	0.2182	Dunnett's multiple comparisons test	stimulation	0.0015
	ctrl vs NI-IO ChR2 50 Hz	7,6	0.9815	0.9752	0.0944	0.1801	Dunnett's multiple comparisons test	stimulation	> 0.9999
	ctrl vs NI ChR2 50 Hz	7,7	0.9815	1.7540	0.0944	0.1963	Dunnett's multiple comparisons test	stimulation	0.0026
	base vs stim NI-MS ChR2 50 Hz	6,6	1.0110	1.6480	0.1646	0.1785	Paired t test	stimulation	0.0196
	base vs stim NI-LH ChR2 50 Hz	7,7	1.0070	1.3540	0.0556	0.1960	Paired t test	stimulation	0.0662
	base vs stim NI-IPN ChR2 50 Hz	5,5	1.0060	1.8670	0.0460	0.2182	Paired t test	stimulation	0.0165
	base vs stim NI ChR2 50 Hz	7,7	1.0060	1.7540	0.1227	0.1963	Paired t test	stimulation	0.0087
7j	ctrl vs NI-MS GtACR1	5,4	0.9087	0.7454	0.0107	0.0656	Mann-Whitney test	inhibition	0.0159
	ctrl vs NI-LH GtACR1	5,5	0.9087	0.8172	0.0107	0.0999	Mann-Whitney test	inhibition	0.6667
	ctrl vs NI-IPN GtACR1	5,4	0.9087	0.9397	0.0107	0.0724	Mann-Whitney test	inhibition	0.8730
	NI vs NI-MS GtACR1	6,4	0.1984	0.7454	0.0532	0.0656	Dunnett's multiple comparisons test	inhibition	< 0.0001
	NI vs NI-LH GtACR1	6,5	0.1984	0.8172	0.0532	0.0999	Dunnett's multiple comparisons test	inhibition	< 0.0001
	NI vs NI-IPN GtACR1	6,4	0.1984	0.9397	0.0532	0.0724	Dunnett's multiple comparisons test	inhibition	< 0.0001
	ctrl vs NI-MS GtACR1	5,4	-1.4020	-20.6000	0.1451	2.7120	Dunnett's multiple comparisons test	inhibition	< 0.0001

7l	ctrl vs NI-LH GtACR1	5,5	-1.4020	-5.8710	0.1451	3.2710	Dunnett's multiple comparisons test	inhibition	0.3509
	ctrl vs NI-IPN GtACR1	5,4	-1.4020	-1.3200	0.1451	1.1980	Dunnett's multiple comparisons test	inhibition	> 0.9999
	NI vs NI-MS GtACR1	6,4	-14.9000	-20.6000	1.6210	2.7120	Dunnett's multiple comparisons test	inhibition	0.2004
	NI vs NI-LH GtACR1	6,5	-14.9000	-5.8710	1.6210	3.2710	Dunnett's multiple comparisons test	inhibition	0.0143
	NI vs NI-IPN GtACR1	6,4	-14.9000	-1.3200	1.6210	1.1980	Dunnett's multiple comparisons test	inhibition	0.0007
7n	ctrl vs NI-MS GtACR1	5,4	1.0100	0.9930	0.0378	0.1461	Unpaired t test	inhibition	0.9048
	ctrl vs NI-LH GtACR1	5,5	1.0100	0.9875	0.0378	0.1004	Unpaired t test	inhibition	0.8408
	ctrl vs NI-IPN GtACR1	5,4	1.0100	0.9899	0.0378	0.1250	Unpaired t test	inhibition	0.8832
	ctrl vs NI GtACR1	5,6	1.0100	0.7355	0.0378	0.0638	Unpaired t test	inhibition	0.0067
	base vs stim, NI GtACR1	6,6	1.0370	0.7355	0.0454	0.0638	Paired t test	inhibition	0.0007
Sup.1d	WT vs Het, NI	3,7	1.0820	0.3911	0.2656	0.0765	Unpaired t test	genotype	0.0085
	WT vs Hom, NI	3,4	1.0820	0.0118	0.2656	0.0062	Unpaired t test	genotype	0.0048
Sup.1e	WT vs Het, OB	3,7	1.1140	0.4090	0.3845	0.0901	Unpaired t test	genotype	0.0312
	WT vs Hom, OB	3,4	1.1140	0.0003	0.3845	0.0003	Unpaired t test	genotype	0.0180
Sup.1f	WT vs Het, locomotor activity	10,16	86.4200	95.3400	5.8560	3.5440	Unpaired t test	genotype	0.3385
	WT vs Hom, locomotor activity	10,11	86.4200	99.1400	5.8560	4.3460	Unpaired t test	genotype	0.1609
	WT vs Het, time in center	10,16	14.9300	15.1100	2.6820	1.3020	Unpaired t test	genotype	0.9976
	WT vs Hom, time in center	10,11	14.9300	15.3100	2.6820	2.4300	Unpaired t test	genotype	0.9916
Sup.2e	rest vs locomotion	7,7	0.2697	11.0773	0.4200	2.8088	Wilcoxon rank sum test	locomotion	0.0006
Sup.2k	correlation coefficient (GC6 and locomotion)	6.0000	0.8512	N/A	0.0340	N/A	one sample t test	N/A	< 0.0001
	correlation coefficient (GC6 and pupil)	6.0000	0.8389	N/A	0.0600	N/A	one sample t test	N/A	< 0.0001
	correlation coefficient (GC6 and theta)	6.0000	0.6959	N/A	0.0610	N/A	one sample t test	N/A	< 0.0001
	correlation coefficient (GC6 and lick)	6.0000	0.2183	N/A	0.1378	N/A	one sample t test	N/A	0.1741
	correlation coefficient (locomotion and pupil)	6.0000	0.7459	N/A	0.0463	N/A	one sample t test	N/A	< 0.0001
	correlation coefficient (locomotion and theta)	6.0000	0.7370	N/A	0.0596	N/A	one sample t test	N/A	< 0.0001
	correlation coefficient (locomotion and lick)	6.0000	-0.0015	N/A	0.1252	N/A	one sample t test	N/A	0.9912
	correlation coefficient (pupil and theta)	6.0000	0.6543	N/A	0.0346	N/A	one sample t test	N/A	< 0.0001
	correlation coefficient (pupil and lick)	6.0000	0.2841	N/A	0.1336	N/A	one sample t test	N/A	0.0868
	correlation coefficient (theta and lick)	6.0000	0.0424	N/A	0.1368	N/A	one sample t test	N/A	0.7691
Sup.4f	ctrl vs GtACR1	6,6	19.1700	100.0000	3.7450	0.0000	Unpaired t test	inhibition	< 0.0001
Sup.4g	ctrl vs GtACR1	6,6	5.5840	1.9310	0.9439	0.6452	Unpaired t test	inhibition	0.0096
Sup.4j	ctrl vs eNpHR3.0	6,6	0.0487	0.0444	0.0072	0.0076	Wilcoxon matched-pairs signed rank test	inhibition	0.4375
Sup.4k	ctrl vs eNpHR3.0	6,6	4.5960	2.3110	0.9062	1.1000	Wilcoxon matched-pairs signed rank test	inhibition	0.0313
Sup.4l	ctrl vs eNpHR3.0	6,6	0.3539	0.3331	0.0301	0.0238	Wilcoxon matched-pairs signed rank test	inhibition	> 0.9999
Sup.5c	rest vs locomotion	7,7	0.5941	17.8400	0.7398	3.6388	Wilcoxon rank sum test	locomotion	0.0006
Sup.5f	ctrl vs GtACR1	7,7	13.4300	1.8570	0.6851	1.8570	Sidak's multiple comparisons test	inhibition	< 0.0001
Sup.5g	ctrl vs GtACR1	7,7	60.7100	100.6000	12.0000	17.8100	Sidak's multiple comparisons test	inhibition	0.0312
Sup.5h	ctrl vs GtACR1	7,7	47.2900	100.6000	12.2177	17.8123	Sidak's multiple comparisons test	inhibition	0.0019
Sup.6g	ctrl vs ChR2 5 Hz	6,9	0.5878	3.6470	0.6362	0.9254	Mann Whitney test	stimulation	0.0176
	ctrl vs ChR2 10 Hz	6,9	-1.2750	6.4240	0.5635	1.6550	Mann Whitney test	stimulation	0.0004
	ctrl vs ChR2 20 Hz	6,9	-0.4727	11.3200	0.7874	2.4670	Mann Whitney test	stimulation	0.0008
	ctrl vs ChR2 50 Hz	6,9	0.0513	17.1800	0.5190	4.4010	Mann Whitney test	stimulation	0.0028
Sup.7a	ctrl vs ChR2 50 Hz	7,16	2.7340	5.1640	0.2976	0.5456	Mann Whitney test	stimulation	0.0037
Sup.7b	light conditioning, ctrl vs. ChR2	6,11	-1.5150	-0.2591	2.5160	2.7650	Tukey's multiple comparisons test	stimulation	> 0.9999
	light conditioning, ctrl vs. GtACR1	6,5	-1.5150	-3.6020	2.5160	3.1640	Tukey's multiple comparisons test	inhibition	0.9999
	opposite, ctrl vs. ChR2	6,11	-3.4830	-0.7727	1.9730	2.7060	Tukey's multiple comparisons test	stimulation	0.9972
	opposite, ctrl vs. GtACR1	6,5	-3.4830	0.2460	1.9730	2.6440	Tukey's multiple comparisons test	inhibition	0.9927
	middle, ctrl vs. ChR2	6,11	4.9980	1.0360	1.8910	1.4430	Tukey's multiple comparisons test	stimulation	0.9675
	middle, ctrl vs. GtACR1	6,5	4.9980	3.3540	1.8910	2.2770	Tukey's multiple comparisons test	inhibition	> 0.9999
Sup.7c	base vs stim, GFP	5,5	407.2000	435.2000	17.6300	32.3700	Paired t test	light	0.2745
	base vs stim, ChR2	8,8	410.4000	381.2000	18.7900	21.2800	Paired t test	stimulation	0.3174
	base vs stim, GtACR1	8,8	427.2000	292.4000	25.0000	62.8100	Paired t test	inhibition	0.0812
Sup.10c	ctrl vs vglut2 ChR2 10 Hz	8,9	0.2256	2.754	0.09237	0.6815	Sidak's multiple comparisons test	stimulation	0.2904
	ctrl vs vgat ChR2 10 Hz	8,8	0.2256	0.5281	0.09237	0.355	Sidak's multiple comparisons test	stimulation	0.9997
	ctrl vs NMB ChR2 10 Hz	8,7	0.2256	8.055	0.09237	1.871	Sidak's multiple comparisons test	stimulation	< 0.0001

	ctrl vs vglut2 ChR2 50 Hz	8,9	0.1525	6.357	0.07544	1.483	Sidak's multiple comparisons test	stimulation	0.0002
	ctrl vs vgat ChR2 50 Hz	8,8	0.1525	0.02443	0.07544	0.01669	Sidak's multiple comparisons test	stimulation	0.9999
	ctrl vs NMB ChR2 50 Hz	8,7	0.1525	10.47	0.07544	1.463	Sidak's multiple comparisons test	stimulation	< 0.0001
Sup.10e	ctrl vs vglut2 ChR2 10 Hz	8,9	99.66	148.1	1.354	9.759	Sidak's multiple comparisons test	stimulation	0.0001
	ctrl vs vgat ChR2 10 Hz	8,8	99.66	102.9	1.354	2.853	Sidak's multiple comparisons test	stimulation	0.9996
	ctrl vs NMB ChR2 10 Hz	8,7	99.66	115.1	1.354	6.334	Sidak's multiple comparisons test	stimulation	0.5771
	ctrl vs vglut2 ChR2 50 Hz	8,9	101.4	164.3	1.231	13.59	Sidak's multiple comparisons test	stimulation	< 0.0001
	ctrl vs vgat ChR2 50 Hz	8,8	101.4	107.6	1.231	3.088	Sidak's multiple comparisons test	stimulation	0.9895
	ctrl vs NMB ChR2 50 Hz	8,7	101.4	132.4	1.231	6.996	Sidak's multiple comparisons test	stimulation	0.0359
Sup.10h	ctrl vs vglut2 ChR2 10 Hz	8,9	5.118	6.869	0.2317	0.5763	Sidak's multiple comparisons test	stimulation	0.0323
	ctrl vs vgat ChR2 10 Hz	8,8	5.118	4.929	0.2317	0.3191	Sidak's multiple comparisons test	stimulation	0.9998
	ctrl vs NMB ChR2 10 Hz	8,7	5.118	7.48	0.2317	0.6653	Sidak's multiple comparisons test	stimulation	0.0033
	ctrl vs vglut2 ChR2 50 Hz	8,9	5.333	7.056	0.3315	0.572	Sidak's multiple comparisons test	stimulation	0.0364
	ctrl vs vgat ChR2 50 Hz	8,8	5.333	4.624	0.3315	0.2625	Sidak's multiple comparisons test	stimulation	0.8357
	ctrl vs NMB ChR2 50 Hz	8,7	5.333	8.182	0.3315	0.2266	Sidak's multiple comparisons test	stimulation	0.0003
Sup.10i	ctrl vs vglut2 ChR2 10 Hz	8,9	0.2374	0.5064	0.02467	0.07379	Sidak's multiple comparisons test	stimulation	0.0013
	ctrl vs vgat ChR2 10 Hz	8,8	0.2374	0.2406	0.02467	0.03346	Sidak's multiple comparisons test	stimulation	> 0.9999
	ctrl vs NMB ChR2 10 Hz	8,7	0.2374	0.5927	0.02467	0.06956	Sidak's multiple comparisons test	stimulation	< 0.0001
	ctrl vs vglut2 ChR2 50 Hz	8,9	0.2922	0.3726	0.02552	0.04536	Sidak's multiple comparisons test	stimulation	0.8117
	ctrl vs vgat ChR2 50 Hz	8,8	0.2922	0.2922	0.02552	0.02552	Sidak's multiple comparisons test	stimulation	> 0.9999
	ctrl vs NMB ChR2 50 Hz	8,7	0.2922	0.5964	0.02552	0.06675	Sidak's multiple comparisons test	stimulation	0.0006
Sup.12d	ctrl vs vglut2 ChR2 10 Hz, speed	5,6	0.5537	6.5420	0.1651	2.0730	Unpaired t test	stimulation	0.0284
	ctrl vs vgat ChR2 10 Hz, speed	5,5	0.5537	1.3540	0.1651	0.4844	Unpaired t test	stimulation	0.1567
	ctrl vs chat ChR2 10 Hz, speed	5,6	0.5537	3.0230	0.1651	0.9969	Unpaired t test	stimulation	0.0535
	ctrl vs vglut2 ChR2 50 Hz, speed	5,6	0.6644	31.4200	0.2768	5.6120	Unpaired t test	stimulation	0.0008
	ctrl vs vgat ChR2 50 Hz, speed	5,5	0.6644	2.6580	0.2768	0.9254	Unpaired t test	stimulation	0.0729
	ctrl vs chat ChR2 50 Hz, speed	5,6	0.6644	8.4290	0.2768	1.3320	Unpaired t test	stimulation	0.0006
Sup.12g	ctrl vs vglut2 ChR2 10 Hz, pupil	5,6	101.0000	130.8000	1.1870	8.1260	Unpaired t test	stimulation	0.0093
	ctrl vs vgat ChR2 10 Hz, pupil	5,5	101.0000	116.5000	1.1870	5.3250	Unpaired t test	stimulation	0.0217
	ctrl vs chat ChR2 10 Hz, pupil	5,6	101.0000	107.1000	1.1870	1.6190	Unpaired t test	stimulation	0.0166
	ctrl vs vglut2 ChR2 50 Hz, pupil	5,6	103.0000	137.9000	1.3830	10.1300	Unpaired t test	stimulation	0.0126
	ctrl vs vgat ChR2 50 Hz, pupil	5,5	103.0000	123.2000	1.3830	9.2980	Unpaired t test	stimulation	0.0633
	ctrl vs chat ChR2 50 Hz, pupil	5,6	103.0000	121.3000	1.3830	4.6960	Unpaired t test	stimulation	0.0075
Sup.12j	ctrl vs vglut2 ChR2 10 Hz, theta freq	5,6	4.6890	8.7440	0.3474	0.6838	Unpaired t test	stimulation	0.0008
	ctrl vs vgat ChR2 10 Hz, theta freq	5,5	4.6890	5.2280	0.3474	0.7392	Unpaired t test	stimulation	0.5277
	ctrl vs chat ChR2 10 Hz, theta freq	5,6	4.6890	4.9830	0.3474	0.2938	Unpaired t test	stimulation	0.5304
	ctrl vs vglut2 ChR2 50 Hz, theta freq	5,6	4.8440	7.8530	0.2804	0.4949	Unpaired t test	stimulation	0.0007
	ctrl vs vgat ChR2 50 Hz, theta freq	5,5	4.8440	5.0800	0.2804	0.5249	Unpaired t test	stimulation	0.7020
	ctrl vs chat ChR2 50 Hz, theta freq	5,6	4.8440	6.4550	0.2804	0.3716	Unpaired t test	stimulation	0.0087
Sup.12k	ctrl vs vglut2 ChR2 10 Hz, theta power	5,6	0.9961	3.4980	0.1617	0.4516	Unpaired t test	stimulation	0.0010
	ctrl vs vgat ChR2 10 Hz, theta power	5,5	0.9961	1.2440	0.1617	0.2307	Unpaired t test	stimulation	0.4054
	ctrl vs chat ChR2 10 Hz, theta power	5,6	0.9961	1.1320	0.1617	0.2254	Unpaired t test	stimulation	0.6487
	ctrl vs vglut2 ChR2 50 Hz, theta power	5,6	0.9905	2.1600	0.1433	0.1986	Unpaired t test	stimulation	0.0013
	ctrl vs vgat ChR2 50 Hz, theta power	5,5	0.9905	0.8341	0.1433	0.2200	Unpaired t test	stimulation	0.5679
	ctrl vs chat ChR2 50 Hz, theta power	5,6	0.9905	1.5290	0.1433	0.1292	Unpaired t test	stimulation	0.0209

Supplementary Table 2. Summary of statistical analyses for *in vitro* electrophysiology									
**Figure #**	**Conditions**	**n per group**	**Group1 Mean±SEM (pA)**		**Group2 Mean± SEM (pA)**		**Analysis**	**Factor**	**P value**
5g	ctrl vs DNQX & gabazine	6, 6	111.5	31.3	6.7	1.3	Wilcoxon rank sum test	DNQX & gabazine	0.0022
	DNQX & gabazine vs wash	6, 5	6.7	1.3	11.6	1.1	Wilcoxon rank sum test	wash	0.0303
5i	ctrl vs DNQX & gabazine	9, 9	278.9	131.7	9.8	1.7	Wilcoxon rank sum test	DNQX & gabazine	<0.0001
	DNQX & gabazine vs wash	9, 7	9.8	1.7	30.9	6.8	Wilcoxon rank sum test	wash	0.0007
5k	ctrl vs DNQX	6, 6	125.4	52.1	6.9	1.7	Wilcoxon rank sum test	DNQX	0.0022
	DNQX vs wash	6, 6	6.9	1.7	37.1	13.2	Wilcoxon rank sum test	wash	0.0411
5m	ctrl vs gabazine	6, 6	232.7	161.6	15.6	6.3	Wilcoxon rank sum test	gabazine	0.0152
	gabazine vs wash	6, 4	15.6	6.3	15.9	4.1	Wilcoxon rank sum test	wash	0.6095
Sup.8j	ctrl vs DNQX & gabazine	2, 2	41.2	4.7	7.6	3.7	Wilcoxon rank sum test	DNQX & gabazine	0.3333
	DNQX & gabazine vs wash	2, 1	7.6	3.7	27.5	0.0	Wilcoxon rank sum test	wash	0.6667
6f	ctrl vs gabazine	9, 9	197.6	69.81	17.84	3.303	Wilcoxon rank sum test	gabazine	0.0039
	gabazine vs wash	9, 8	17.84	3.303	38.98	16.75	Wilcoxon rank sum test	wash	0.3828
6h	ctrl vs gabazine	5, 5	119.1	79.2	8.3	4.4	Wilcoxon rank sum test	gabazine	0.0317
	gabazine vs wash	5, 4	8.3	4.4	22.2	3.5	Wilcoxon rank sum test	wash	0.1111
Sup.9h	ctrl vs DNQX	10, 10	157.7	44.27	15.54	2.053	Wilcoxon rank sum test	gabazine	0.002
	DNQX vs wash	10, 9	15.54	2.053	32.93	8.14	Wilcoxon rank sum test	wash	0.0078
6j	ctrl vs gabazine	8, 8	202.8	56.01	20.0	2.986	Wilcoxon rank sum test	gabazine	0.0078
	gabazine vs wash	8, 6	20.0	2.986	36.74	12.74	Wilcoxon rank sum test	wash	0.2188

Supplementary Table 3. Summary of reagents and mouse lines		
**Antibodies and probes**		
anti-Rln3	R&D Systems	Cat# AF3107
anti-CRFR1	Aviva System Biology	Cat# OAEB02329
anti-GFP	Thermo Fisher Scientific	Cat# A-11122
anti-RFP	Abcam	Cat# ab62341
anti-VGAT probe	Advanced Cell Diagnostics	Cat# 319191
anti-NMB probe	Advanced Cell Diagnostics	Cat# 459931
**Drugs**		
Gabazine	Sigma-Aldrich	Cat# S106-10MG
DNQX	Sigma-Aldrich	Cat# D0540-25MG
TTX	Tocris	Cat# 1078
4-AP	Sigma-Aldrich	Cat# A78403
muscimol	Tocris	Cat# 0289
clonidine hydrochloride	Sigma-Aldrich	Cat# 4205-91-8
pancuronium hydrochloride	Sigma-Aldrich	Cat# P1918-10MG
**Virus vectors**		
AAV9-DIO-ChR2-mCherry	Our lab	Addgene plasmid #20297
AAV9-DIO-mCherry	Our lab	N/A
AAV9-DIO-EmGFP	Our lab	Addgene Plasmid #14757
AAV9-DIO-GCaMP6m	Our lab	Addgene Plasmid #40754
AAV9-DIO-GtACR1-P2A-GFP	Our lab	N/A
AAV9-DIO-TVA-mCherry	Our lab	Addgene Plasmid #38044
AAV9-DIO-RVG	Our lab	Addgene Plasmid #48333
SAD-ΔG- EmGFP	BrainVTA Inco (Wuhan, China)	N/A
AAV9-DIO-tdTomato-T2A-SypEGFP	Shanghai Taitool Bioscience	N/A
AAV-retro-CAG-DIO-Flp	Shanghai Taitool Bioscience	N/A
AAV-retro-DIO-mGFP	Shanghai Taitool Bioscience	N/A
AAV-retro-DIO-mCherry	Shanghai Taitool Bioscience	N/A
AAV9-fDIO-ChR2-mCherry	Shanghai Taitool Bioscience	N/A
AAV9-DIO-eNpHR3.0-EYFP	Shanghai Taitool Bioscience	N/A
**Mouse lines**		
NMB-Cre	This paper	
Vgat-ires-Cre	Jackson Laboratory	Stock NO: 028862
Vglut2-ires-Cre	Jackson Laboratory	Stock NO: 016963
Chat-Cre	MMRRC	MGI:3836624
