# Supplementary material for: Control of locomotor speed, arousal, and hippocampal theta rhythms by the nucleus incertus
Source: Nat Commun. 2020 Jan 14;11:262. doi: 10.1038/s41467-019-14116-y (PMC6959274; doi:10.1038/s41467-019-14116-y)
Supplement: Supplementary file 2 — Reporting Summary [file 41467_2019_14116_MOESM2_ESM.pdf]

nature research

Corresponding author(s): Minmin Luo

Last updated by author(s): Dec 11, 2019

Reporting Summary

Nature Research wishes to improve the reproducibility of the work that we publish. This form provides structure for consistency and transparency in reporting. For further information on Nature Research policies, see Authors & Referees and the Editorial Policy Checklist .

Statistics

For all statistical analyses, confirm that the following items are present in the figure legend, table legend, main text, or Methods section.

n/a	Confirmed	
☐	☒	The exact sample size (n) for each experimental group/condition, given as a discrete number and unit of measurement
☐	☒	A statement on whether measurements were taken from distinct samples or whether the same sample was measured repeatedly
☐	☒	The statistical test(s) used AND whether they are one- or two-sided<br>*Only common tests should be described solely by name; describe more complex techniques in the Methods section.*
☒	☐	A description of all covariates tested
☐	☒	A description of any assumptions or corrections, such as tests of normality and adjustment for multiple comparisons
☐	☒	A full description of the statistical parameters including central tendency (e.g. means) or other basic estimates (e.g. regression coefficient) AND variation (e.g. standard deviation) or associated estimates of uncertainty (e.g. confidence intervals)
☐	☒	For null hypothesis testing, the test statistic (e.g. F, t, r) with confidence intervals, effect sizes, degrees of freedom and P value noted<br>*Give P values as exact values whenever suitable.*
☒	☐	For Bayesian analysis, information on the choice of priors and Markov chain Monte Carlo settings
☒	☐	For hierarchical and complex designs, identification of the appropriate level for tests and full reporting of outcomes
☒	☐	Estimates of effect sizes (e.g. Cohen's d, Pearson's r), indicating how they were calculated

Our web collection on statistics for biologists contains articles on many of the points above.

Software and code

Policy information about availability of computer code

Data collection	USB-6008 DAQ (National Instrument); OpenEphys board (http://www.open-ephys.org/); Power3-1401(CED); MultiClamp 700B amplifier (Molecular Devices); DigiData 1440 (Molecular Devices); Fiber recording system (THINKERTECH); Infrared camera(Wei Xin Shi Jie); Automated slide scanner (VS120 Virtual Slide, Olympus); Confocal microscope (Nikon A1)
Data analysis	Matlab (version R2016b); GraphPad Prism (7.0a); ImageJ (FIJI) (version 2.0.0)

For manuscripts utilizing custom algorithms or software that are central to the research but not yet described in published literature, software must be made available to editors/reviewers. We strongly encourage code deposition in a community repository (e.g. GitHub). See the Nature Research guidelines for submitting code & software for further information.

Data

Policy information about availability of data

All manuscripts must include a data availability statement. This statement should provide the following information, where applicable:

- Accession codes, unique identifiers, or web links for publicly available datasets
- A list of figures that have associated raw data
- A description of any restrictions on data availability

The data that support the findings of this study are available from the corresponding author upon reasonable request.

Field-specific reporting

Please select the one below that is the best fit for your research. If you are not sure, read the appropriate sections before making your selection.

Life sciences study design

All studies must disclose on these points even when the disclosure is negative.

Sample size	No hypothesis based experiment was performed. Therefore, the sample size was not pre-determined. Previous experiments in our laboratory suggest that sample sizes of 4–7 animals (biological replicates) for each experimental condition are sufficient to detect behavioral effects elicited with optogenetics.
Data exclusions	No data were excluded, except in cases where videos with poor readout quality (e.g. due to the mouse not fully opening its eyes) or LFP with noise.
Replication	All experiments were independently performed >= 3 times with similar results, except the control experiments of RV tracing depicted in Supplementary Fig. 8b-d.
Randomization	Mice were randomly distributed into given groups after matching for age. Where possible, stimulation protocols were applied in pseudorandom order generated by Matlab.
Blinding	Experimenters were not 'blinded' to group allocations. All animals (control and experiment groups) in fiber photometry recording and optogenetics manipulation experiments were prepared and performed in the same condition and analyzed with custom Matlab program.

Reporting for specific materials, systems and methods

We require information from authors about some types of materials, experimental systems and methods used in many studies. Here, indicate whether each material, system or method listed is relevant to your study. If you are not sure if a list item applies to your research, read the appropriate section before selecting a response.

Materials & experimental systems

n/a	Involved in the study	
☐	☒	Antibodies
☒	☐	Eukaryotic cell lines
☒	☐	Palaeontology
☐	☒	Animals and other organisms
☒	☐	Human research participants
☒	☐	Clinical data

Methods

n/a	Involved in the study	
☒	☐	ChIP-seq
☒	☐	Flow cytometry
☒	☐	MRI-based neuroimaging

Antibodies

Antibodies used	The following primary antibodies were used in this study: anti-Rln3 (R&D Systems, Cat# AF3107), anti-CRFR1 (Aviva System, Biology, Cat# OAEB02329), anti-GFP (Thermo Fisher Scientific, Cat# A-11122), anti-RFP (Abcam, Cat# ab62341)<br>The following secondary antibodies were used in this study: Donkey anti-goat (705-065-147, 705-065-147), Goat anti-rabbit (111-165-008, 111-545-144) from Jackson ImmunoResearch.
Validation	These antibodies were extensively used in previous studies. Staining pattern was compared to existing literature when possible. Details of antibodies and their uses can be found in Methods section and Supplementary Table 3.

Animals and other organisms

Policy information about studies involving animals; ARRIVE guidelines recommended for reporting animal research

Laboratory animals	NMB-Cre heterozygous mice were maintained on a mixed FVB/N & C57BL/6N background. Adult (8-16 weeks old) Vgat-ires-Cre mice [STOCK B6J.129S6(FVB)-Slc32a1tm2(cre)Lowl/MwarJ, NO: 028862] and Vglut2-ires-Cre mice [STOCK Slc17a6tm2(cre)Lowl/J, NO: 016963] of were obtained from the Jackson Laboratory (USA). The Chat-Cre transgenic mice [Tg (ChAT-Cre)24Gsat] were provided by MMRRC (Davis, CA, USA). Wildtype C57BL6/N mice were purchased from VitalRiver (Beijing, China).
Wild animals	The study did not involve wild animals.
Field-collected samples	The study did not involve samples collected from the field.
Ethics oversight	All procedures were conducted with the approval of the Animal Care and Use Committee of the National Institute of Biological Sciences, Beijing, in accordance with governmental regulations of China.

Note that full information on the approval of the study protocol must also be provided in the manuscript.
