# Supplementary material for: Control of locomotor speed, arousal, and hippocampal theta rhythms by the nucleus incertus
Source: Nat Commun. 2020 Jan 14;11:262. doi: 10.1038/s41467-019-14116-y (PMC6959274; doi:10.1038/s41467-019-14116-y)
Supplement: Supplementary file 3 — Description of Additional Supplementary Files [file 41467_2019_14116_MOESM3_ESM.pdf]

**Description of Additional Supplementary Files**

File Name: Supplementary Movie 1
Description: The activity of NI NMB neurons is correlated with locomotor speed, pupil-linked arousal level, and theta rhythm power. Top panel shows images captured from the eye that was ipsilateral to the craniotomy. Green trace corresponds to locomotor speed. First black trace corresponds to the GCaMP signals of the NI NMB neurons. Second black trace corresponds to the hippocampal LFP signals.

File Name: Supplementary Movie 2
Description: Inhibiting NI NMB neurons rapidly and reversibly suppresses locomotion, arousal, and theta rhythms. Top image corresponds to image captured from the eye that was ipsilateral to the craniotomy. The timing of delivery of continuous light (5 s) was indicated in the northwest of the image. Green trace corresponds to locomotor speed. Black trace corresponds to the hippocampal LFP signals.

File Name: Supplementary Movie 3
Description: Optogenetic inhibition of NI NMB neurons disrupts animal chasing for food. A GtACR1-expressing mouse was trained to chase a moving food tray (20 cm·s -1) to retrieve food pellets. In the inhibition session, we briefly delivered light pulses (~2 s duration; labeled "light inhibition") into the NI when the mouse walked close to the food tray. The optogenetic inhibition slowed down locomotion and completely abolished animal's ability to collect food from the moving food tray. Mice often walked toward walls following the termination of optogenetic inhibition, a result suggesting thigmotaxis. Shining light into the NI of EmGFP-expressing control mice does not disrupt food chasing

File Name: Supplementary Movie 4
Description: Optogenetic stimulation of NI NMB neurons induces locomotion, increases arousal level, and promotes hippocampal theta power. Top image corresponds to image captured from the eye that was ipsilateral to the craniotomy. The timing of delivery of light pulses (a train of pulses with 5 ms width at 50 Hz, 5 s) was indicated in the northwest of the image. Green trace corresponds to locomotor speed. Black trace corresponds to the hippocampal LFP signals.
